# Transcriptomic signatures of rare variant impacts across sex and the X chromosome

**DOI:** 10.1016/j.xhgg.2025.100463

**Published:** 2025-05-31

**Authors:** Rachel A. Ungar, Taibo Li, Nikolai G. Vetr, Nicole Ersaro, Alexis Battle, Stephen B. Montgomery

**Affiliations:** 1 Department of Genetics, School of Medicine, Stanford University, Stanford, CA, USA; 2 Department of Pathology, School of Medicine, Stanford University, Stanford, CA, USA; 3 Department of Biomedical Engineering, Johns Hopkins University, Baltimore, MD, USA; 4 Department of Biomedical Data Science, School of Medicine, Stanford University, Stanford, CA, USA; 5 Department of Computer Science, Johns Hopkins University, Baltimore, MD, USA

**Keywords:** rare variant, X chromosome, machine learning, sex differences

## Abstract

The human X chromosome contains hundreds of genes and has well-established impacts on sex differences and traits. However, the X chromosome is often excluded from many genetic analyses, limiting broader understanding of variant effects. In particular, the functional impact of rare variants on the X chromosome is understudied. To investigate functional rare variants on the X chromosome, we use observations of outlier gene expression from Genotype Tissue Expression consortium data. We show that outlier genes are enriched for having nearby rare variants on the X chromosome, and this enrichment is stronger for males. Using the RIVER model, we identified 733 rare variants in 450 genes predicted to have functional differences between males and females. We examined the pharmacogenetic implications of these variants and observed that 25% of drugs with a known sex difference in adverse drug reactions were connected to genes that contained a sex-biased rare variant. We further identify that sex-biased rare variants preferentially impact transcription factors with predicted sex-differential binding, such as the *XIST*-modulated *SIX1*. Overall, we observed more within-sex variation than between-sex variation. Combined, our study investigates functional rare variants on the X chromosome, and further details how sex stratification of variant effect prediction improves identification of rare variants with predicted sex-biased effects, transcription factor biology, and pharmacogenomic impacts.

## Teaser

This paper examines the impact of rare variants and sex on gene expression across the X chromosome and autosomes, ultimately identifying over 700 rare variants with predicted functional differences between males and females. Their role in transcription factor binding and pharmacogenetics is further explored.

## Introduction

The exclusion of the X chromosome in genetic studies remains prevalent despite its role in sex determination and human traits. Only one-quarter of current genome-wide association studies (GWASs) include the X chromosome, yet it contains 800 genes, over 60% of which have been linked to genetic disorders.^1^^,^^2^ Unlike other chromosomes, the X chromosome differs in copy number between males and females and is also subject to aneuploidy, with up to five copies being reported in humans.^3^^,^^4^ The difference in copy number between males and females has been suggested as a potential mechanism for differences in traits and disease between the sexes.^4^

Sex-biased genes are located across both the autosomes and X chromosome, but a larger role has been implicated for these genes on the X chromosome.^5^^,^^6^ Across metazoans, 30%–60% of genes are estimated to be sex biased and approximately 37% of human genes are sex biased in at least one tissue, although with predominantly small effect sizes.^5^^,^^7^ Biological sex can lead to differences in disease risk and even impact drug efficacy and susceptibility to adverse drug reactions.^4^^,^^8^^,^^9^

Even among genetic studies that consider sex and the X chromosome, most have been restricted to common variants. For example, when the X chromosome is included in analyses of molecular traits such as gene expression, a small number of expression quantitative trait loci (eQTLs) are often discovered due to unique selective pressures on the X chromosome.^10^^,^^11^ Historically few sex-biased eQTLs have been discovered from bulk RNA-seq, likely due to power issues.^5^^,^^12^^,^^13^ As eQTL studies focus on common variants, there remains an ongoing gap in understanding how low-frequency or rare variants may contribute to sex differences in gene expression and, ultimately, complex traits and diseases.

Rare variants, by definition, each occur in few people in the general population (allele frequency < 1%). However, collectively they are common with singletons, alleles seen in just one individual, the most abundant and impactful class of variants.^14^^,^^15^ Despite their importance, the study of rare variants cannot easily follow traditional common-variant approaches, such as GWASs, as both multiple-testing burden and sample size required for adequate power impede their analysis. In lieu of association testing, understanding the functional properties of impactful rare variants allows for effective prediction of a functional effect. Prior studies have employed an outlier-based approach to identify functional properties of rare variants impacting extreme gene expression.^16^^,^^17^^,^^18^^,^^19^^,^^20^^,^^21^^,^^22^ This approach is analogous to efforts to study extreme phenotypes in population samples to enrich for impactful rare variants.^23^^,^^24^ However, it has yet to be applied to the X chromosome and across sexes where dosage and sex-specific regulation can impact discovery.

In our study, we evaluated outlier-associated rare variants on the X chromosome, and further applied a sex-stratified approach genome wide across the X chromosome and the autosomes. Sex stratification has repeatedly been shown to impact discovery in genomic analyses.^25^ Despite advances in machine learning and AI-based variant effect prediction, sex-specific datasets have received limited attention. The same applies to rare variant prioritization methods such as RIVER that integrate molecular outlier data.^22^ Using the Genotype Tissue Expression (GTEx) consortium data, we detected multi-tissue outliers on the X chromosome, investigated the properties of outlier-enriched rare variants, and the impact of sex stratification on training and prediction. We subsequently sex-stratified GTEx data, trained and applied the RIVER model to prioritize rare variants with sex-specific functional effects, and examined their potential interactions with drugs and sex-specific transcription factors (TFs). Combined, this work highlights rare variant effects on the X chromosome and the value of sex-informed analyses of variant effects.

## Methods

### Variant filtering and annotation

Variants were obtained from whole-genome sequencing from the GTEx v.8 dataset.^26^ Allele frequencies were then annotated for each site using gnomAD v.3.0.^14^^,^^27^ Following Ferraro et al., individuals were subset to only include those with high European ancestry (genetic PC1 < 0 and PC2 > 0.05, where PC stands for principal component) due to the low proportion of other ancestries and subsequent smaller group sizes when conducting sex stratification by ancestry.^21^ We further excluded any individuals with atypical sex chromosomes.^26^ We considered only rare variants within genic regions and at most +/−5 kb from a gene. We restricted our analysis to lncRNA and protein-coding genes using GENCODEv26.^28^ All variants were subsequently annotated with combined annotation dependent depletion (CADD) scores.^29^ For the X chromosome, we performed additional variant filtering. First, we removed variants in ENCODE blacklisted regions^30^ (Figure S1). We collapsed this to just one variant per gene, selecting the rarest variant that passed a given CADD threshold of 0 or 15. This led to identifying over a million unique gene-variant pairs at a MAF < 0.001 (Table 1).

**Table 1:** Number of gene-variant pairs across minor allele frequency bins

MAF range	Autosomes	X chromosome
0–0.0001	596,184	10,783
0–0.001	1,090,188	20,471
0–0.01	1,556,156	31,237
0–0.05	1,754,935	35,529
0–0.10	1,812,921	36,676
0.001–0.01	465,968	10,766
0.01–0.05	198,778	4,292
0.05–0.10	57,982	1,147

### Sex stratification of GTEx samples

The number of copies of the X chromosome is one of the components that define the biological sex of an individual. The biological term sex is a multidimensional concept referring to a combination of sex chromosomes, internal and external genitalia, and sex hormones.^31^ In this paper, we used a narrow definition of sex, in which we only considered sex chromosomes, and referred to individuals with XX chromosomes as female and XY chromosomes as male.

For outlier analyses, we stratified our samples into four separate groups: female (F), male (M), combined (all), combined (equal sample size). Sex stratification was done prior to read count filtering. We defined samples as male based on the presence of XY sex chromosomes and the GTEx-reported sex, and females based on the presence of XX sex chromosomes and the GTEx-reported sex. As GTEx is male biased, we first aimed to control for power differences by subsetting the male group to be of equal sample size with the female group (N_F_ = N_M_). Subsequently, the combined (all) group was created by taking all samples (N_F_ + N_M_) while the combined (equal sample size) group was created to be of equal sample size (½N_F_ + ½N_M_ = N_B_). The individuals with the maximum number of tissues were included to improve subsequent multi-tissue outlier discovery. Therefore the number of males and females were of equal sample size per tissue for the sex-stratified group. Across various tissues, this resulted in the inclusion of 429 males and 281 females in the sex-stratified group and 276 individuals (133 female, 143 male) in the combined equal sample size group. In the combined all group there were 746 individuals (281 female, 465 male). Null sets were created by shuffling sex labels.

### Expression processing, correction, and outlier calling

Transcripts per million (TPM) values and read counts were obtained from the GTEx v.8 dataset.^26^ We filtered to 22,189 protein-coding and 783 lncRNA genes on the autosomes and X chromosome.

Expression data were processed following the same methods as Ferraro et al.^21^ Briefly, read counts are filtered such that, in the combined group, at least 20% of individuals in at least one sex have a TPM > 0.1 and read count >6, and 5% of individuals do not have zero read counts. Then, for each group, these counts are log transformed, centered, and scaled to create *Z* scores. For each tissue and group, probabilistic estimation of expression residuals (PEER) factors were calculated.^32^ For each tissue and group, corrected counts were calculated using a linear model incorporating the top 3 genetic PCs, top eQTLs, and PEER factors. We regressed sex from these covariates to ensure that sex would not be corrected out before applying these covariates. The residuals of this regression were then transformed into *Z* scores to be used for the outlier analysis. These *Z* scores represent, for a given gene, how far the expression value of one individual is from the mean of all the other individuals. We determined an outlier as any gene-individual pair with an absolute *Z* score greater than 2.5.

Thresholds for multi-tissue outlier discovery were chosen based on parameterizing across a range of *Z* scores and tissues (Figures S2A and S2B). Multi-tissue outliers were required to be outliers in at least three tissues, and the absolute median *Z* score greater than 2.5 across all tissues. Any gene-individual pairs identified as a multi-tissue outlier for more than two individuals were removed.

### Variant relative risks for expression outliers

Enrichment of rare variants in gene expression outliers were calculated in R using epitools.^33^ For this analysis, rare variants were first collapsed to the gene level to assess the presence of any rare variant which also satisfied any additional annotation criteria. Considered annotations were whether there was a variant with greater than or equal to a CADD threshold (in practice this is 0 or 15) and within a given distance (5 kb) from the gene start and end (Figure S2C). If multiple variants passed the annotation and frequency filters, we selected the variant with the lowest minor allele frequency. We defined rareness as a binary (rare, not rare) at different allele frequency thresholds (0.0001, 0.001, 0.01, 0.05, 0.1) across annotations (e.g., CADD ≥ 15) for our relative risk calculations.

For relative risk calculations, we assessed the proportion of outliers with a threshold-passing variant compared with non-outliers.

$$\frac{\frac{outliers\;with\;variant}{total\;outliers}}{\frac{non-outliers\;with\;variant}{total\;non-outliers}}$$

A relative risk score greater than one indicated that there was an enrichment for outliers to have a threshold-passing variant compared with non-outliers, a score less than one indicated that outliers were depleted of variants at a given allele frequency.

### Sex-specific rare variant discovery

To assess the functional impact of rare variants on gene expression, we applied RIVER, a Bayesian hierarchical model that integrates genomic annotations and observed outlier effects.^22^ For model training, we used a set of *n* = 30 genomic annotations calculated from Variant Effect Predictor (VEP), CADD, and genomic locations summarized across all rare variants (MAF < 0.01) within 10 kb of the gene (G layer), and used median *Z* score across tissues estimated from a balanced set of female and male individuals as observed outlier signals (E layer). Binary outlier threshold was set at *p* < 0.05 (corresponding to approximate |Z| > 2, --pvalue_threshold = 0.05). Approximately 1% of gene-individual pairs reached this threshold, which we used for model evaluation (--pvalue_fraction = 0.01). We next separately modeled over- and under-outliers as previous work has identified different categories of rare variants contributing to these signals.^21^ To enrich for outlier signals with genetic effects, we filtered out genes which do not have any outlier individuals (defined as |Z| > 2).

For model evaluation, we leveraged pairs of individuals with the same set of rare variants nearby the same gene (“N2 pair”), where we inferred the regulatory status of the second individual based on genomic annotations and observed outlier status of the first individual. Area under the precision-recall (P-R) curve was used as the evaluation metric. Importantly, these N2 pairs were not included in model training. In total, we had 532,975 instances of (gene, individual), of which we had 38,263 N2 pair individuals for evaluation of the model. RIVER scores range between 0 and 1 in magnitude, where 1 indicates a high probability that the variant drives an outlier expression effect. The sign of the score represents the direction of this effect; negative scores mean a variant reduces expression and positive scores mean a variant increases expression.

To estimate sex-specific effects of rare variants, we trained on both sexes, and then conducted predictions within each sex, thereby obtaining posterior probabilities [P(Z |G, E)] of driving outlier expression levels of nearby genes for each variant in each sex. We also trained a model using expression data on the X chromosome from both sexes, and estimated sex-specific posterior effects similarly. For variants appearing in at least one female and one male, we calculated the median posterior within either females or males to derive the sex-specific posteriors. We defined a variant as sex biased if the posterior probability in one sex was higher than the posterior probability in the other (at a threshold of 0.2). We further classified sex-specific variants based on direction of effect, where a female under-expression rare variant had a significantly higher posterior for under-expression in females than in males (|female posterior| - |male posterior| > 0.2 and female posterior < 0), and similarly for the other three categories.

### Annotation of sex-specific rare variants and gene set enrichment analysis

For each group of sex-biased rare variants, we assessed their overlap with existing annotations by computing the fraction of variants annotated in each VEP category.^34^ For each variant type, we evaluated if the number of sex-biased functional rare variants that had a given annotation compared with the number without this annotation was significantly different than that of non-functional rare variants using a Fisher’s exact test. This was done for male-biased, female-biased, and non sex-biased functional rare variants, and additionally split for those predicted to increase and reduce expression.

To characterize genes associated with sex-specific rare variants, we took all genes predicted by RIVER to be disrupted by these rare variants. Importantly, for rare variants nearby multiple genes, we only took genes for which RIVER posterior showed sex-specific effects as defined above. We grouped female-biased variants as those with higher posterior in females (female over-expression and male under-expression), and similarly defined male-biased variants as those with higher posteriors in males (male over-expression and female under-expression) at a difference greater than 0.2. We then used the clusterProfiler package in R to assess significantly enriched GO terms in female and male-biased gene sets and performed FDR correction on the resulting *p* values.^35^ To assess significance of overlap between genes with a sex-biased rare variant and genes with sex-biased eQTLs reported in GTEx we applied a hypergeometric test.^5^

### Pharmacogenetic annotation and analysis

Genes with sex-biased rare variants predicted by RIVER were linked to drugs using the Drug Gene Interaction database (DGIdb).^36^ A list of drugs with adverse drug reactions was obtained from the Table of Pharmacogenomic Biomarkers in Drug Labeling on the FDA Center for Drug Evaluation and Research website.^37^ A list of sex-biased adverse drug reactions was obtained from Zucker and Prendergast.^38^ The relative risk of the proportion of sex-biased rare variants within a given database compared with non-biased functional rare variants was evaluated using the epitab function using the Wald test from the epitools package.^33^

### Sex-specific TF network analysis

We performed two analyses to investigate the relationship between prioritized rare variants and TF binding. First, we performed a genome-wide motif enrichment analysis using HOMER around 100 bp up and downstream of each class of variants, setting “-size given” and masking repeat regions with all other parameters as default.^39^ We obtained the top enriched motifs where the fraction of motifs overlapping the genomic regions centered on sex-specific rare variants are significantly higher than that overlapping with the background, and highlighted top enriched motifs ranked by enrichment *p* value. Next, for all rare variants within annotated regulatory regions, we estimated disruption of TF binding using FABIAN variant, which leverages a large collection of motif databases to model alterations in TF binding between reference and alternative alleles, across all TFs in the database.^40^ For each variant and each TF, we summarized predicted effects across all models to obtain final effect estimates. To investigate sex-specific TF effect on target genes, we designed metric to combine predicted rare variant effect on TF binding and the posterior estimates of a rare variant underlying expression outlier signals of a nearby gene, where for TF i and gene j, we calculate the regulatory score across SNPs (v) in the 10 kb window as follows

$$\frac{\sum_{v}s_{iv}\;*\;p_{vj}}{\sum_{v,i}s_{iv}\;*\;p_{vj}}$$

where s_iv_ is the predicted disruption of TF i’s motif by variant v, and p_vj_ is the RIVER posterior of variant v on gene j. The denominator is in place to compare relative regulatory effects of all TFs for each gene.

To control for false positive discovery of sex-specific variants, we randomly permuted sex labels in *Z* score calculation and outlier calling five times, and recalculated rare variant posteriors by RIVER. We repeated the same motif enrichment and binding disruption analysis, and only kept top TFs with higher enrichment and estimated binding effects than results from all permuted models.

## Results

### Individuals carry multi-tissue gene expression outliers on the X chromosome

**Figure 1:**
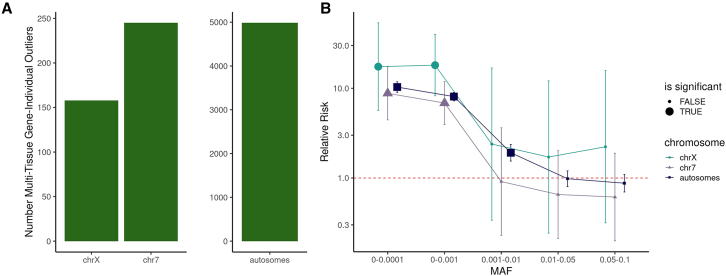
Outliers and rare variants across the autosomes and X chromosome (A) The number of multi-tissue gene-individual outliers for all individuals (both sexes) across the X chromosome, chromosome 7, and the autosomes. (B) The relative risk of an outlier having a nearby variant compared with a non-outlier across different minor allele frequency (MAF) bins for the X chromosome, chromosome 7, and autosomes. Significant enrichments have bigger point sizes. The error bars represent the lower and upper bounds of the 95% confidence interval, and the *p* value is determined by Fisher's exact test with Benjamini-Hochberg (BH) multiple testing correction.

Gene expression outliers can inform likely disease-causing genes and prioritize functional rare variants.^18^^,^^21^ We focused on gene-individual pairs that were outliers (absolute *Z* score > 2.5) in multiple tissues across both sexes (multi-tissue outliers combined sex group, see methods). We identified multi-tissue outliers on the autosomes and X chromosome, and selected chromosome 7 (chr7) as a control for outlier discovery given it has a similar number of genes as the X chromosome. In total, we identified 158 multi-tissue outliers (gene-individual pairs) on the X chromosome, 245 on chr7, and 4,985 on the autosomes (Figure 1A). This corresponded to an average number of gene expression outliers per individual of 0.21 multi-tissue outliers on the X, 0.33 multi-tissue outliers on chr7, and 6.68 multi-tissue outliers across all the autosomes.

Multi-tissue outliers were discovered on the X for 133 unique genes across 135 individuals (18% of total genes tested on the X chromosome) (Figure 1A). Of these genes, 22 have prior evidence of trait relationships in Open Targets with evidence scores >0.6. We evaluated if genes harboring outliers reflected other metrics of selective constraint. Overall, there were more over-expression outliers than under-expression outliers on the X chromosome, consistent with previous studies of autosomes and reflecting higher tolerance for gain-of-expression effects (Figure S3A).^22^ The proportion of over-expression outliers on the X chromosome was significantly higher than in the autosomes (*p* = 0.02).

In gnomAD v.3, constraint metrics for genes have been calculated to identify their tolerance for having missense, synonymous, and loss-of-function variants.^41^^,^^42^^,^^43^ Among these metrics, outlier genes were less tolerant of synonymous mutations (Figure S3B). However, outliers were depleted for genes less tolerant of missense and loss-of-function mutations, which could be expected due to the largely non-disease status of the GTEx samples and cohort (Figure S31B). We also applied a dosage constraint metric using analysis of expression variation (ANEVA) to classify the amount of variation a gene expression is expected to have, and analogously the map of dosage sensitivy (MoD) score to calculate the amount of gene dosage tolerance, where a higher number in each score demonstrates more tolerance.^44^^,^^45^ We observed that genes with higher average expression variation were more likely to be multi-tissue over-expression outliers (Figure S3B).

### Outliers are enriched for rare variants on the X chromosome

Previous studies have reported enrichment of rare variants near multi-tissue gene expression outliers of autosomal genes.^21^^,^^22^ To conduct X chromosome analyses required additional quality control, and variant filtration (see methods).^46^ We subsequently tested for corresponding enrichment of rare variants on the X chromosome across different frequency bins by identifying what proportion of multi-tissue outliers had nearby rare variants compared with the proportion of non-outliers with a nearby rare variant. We observed significant enrichment of gene-proximal rare variants with minor frequencies less than 0.1%, while common variants were not enriched (Figure 1B). Enrichment of rare variants were largely driven by under-expression outliers (Figure S4). We observed the point estimate on the X chromosome to be higher than on the autosomes and chr7; however, this was not significant (subset to be of equal size to chrX).

We next investigated if outliers were enriched for rare variants (MAF < 0.01) within specific regions. For the X chromosome, we observed that more outliers had a rare variant in either a VEP-annotated TF binding sites, upstream gene, regulatory region, 3′ untranslated region, and 5′ untranslated region compared with non-outliers (Figure S5A). These enrichments were seen on the autosomes, but were not significant on chr7 when considered alone (Figures S5B and S5C).

### Estimated impact of sex stratification on outlier discovery

Given that females carry two X chromosomes while males carry just one, we aimed to estimate the impact of sex on outlier discovery. We reasoned that sex stratification could impact outlier discovery in the following ways: a gene could be an outlier in a sex-stratified group but not in the combined group, a gene could be an outlier in a combined group but not in the sex-stratified group, a gene could be an outlier in both the combined and sex-stratified groups but at different strengths, or there could be no substantial impact. First, we simulated how sex stratification could impact outlier discovery.

To simulate the impact of sex stratification, we assumed that males and females were drawn from a normal distribution, and the combined group was drawn from a mixture model composed of these two aforementioned distributions. We then calculated outliers for *sex-stratified* groups (males or females separately) of equal sample size, and then created a subset *sex-combined* group (males and females together) of sample size equal to the sex-stratified groups. We next asked how a shift in mean between the male and female distributions for a given gene would impact outlier discovery assuming constant variance of 1. For a change in the normalized mean of 1 between males and females, approximately 1% of genes will either lose or gain outlier status. The larger the difference in the means between males and females, the more outliers were identified in the sex-stratified groups. Further, we identified outliers in the sex-combined group that were not outliers in the sex-stratified group. We observed this effect up to a mean difference of 1.9 where beyond this point the variance in the sex-combined distribution is large enough that any value called an outlier in the combined group would also be considered an outlier in the sex-stratified group (Figure S6A).

We estimated how a shift in variance impacts outlier calling assuming a constant mean for both distributions of 0, and setting the variance of one distribution to be fixed at 1. The change in proportion of outliers due to a difference in variance was a fixed proportion; for example, if the variance of the male distribution is 3 and the variance of the female distribution is 6 this was equivalent to if the variances were 1 and 2, respectively (Figure S6B). The proportion of outliers lost from the sex-combined group and gained in the sex-stratified group increased as the difference in variance increased, but the proportion of outliers lost happened at a larger rate than the proportion of outliers gained.

### Sex masks gene expression outlier discovery and impacts variant enrichments

**Figure 2:**
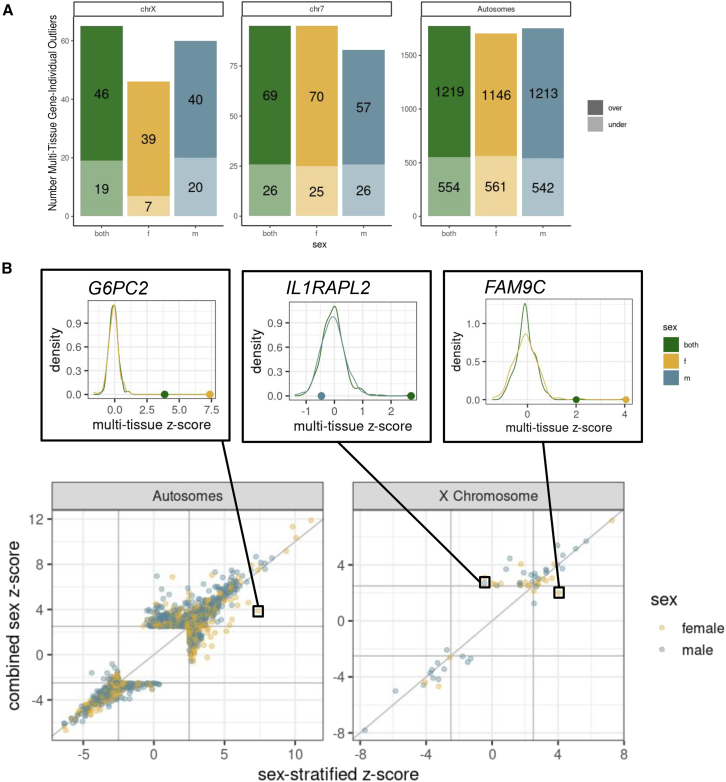
Impact of sex stratification on outlier detection (A) The number of multi-tissue gene-individual outliers across the X chromosomes, chromosome 7, and autosomes. Under-expression outliers are lighter and on the bottom, over-expression outliers are darker and on the top. (B) The median *Z* score across tissues of a gene-individual pair in the sex-stratified group (male or female) compared with the combined sex (both) group for gene-individual pairs that are a multi-tissue outlier in either the sex-stratified or sex-combined group. The individual-gene pair points are colored by their sex. Three examples—G6PC2, IL1RAPL2, and FAM9C—are highlighted above, with the sex-stratified and combined-sex *Z* score multi-tissue *Z* scores distribution across all individuals for that given gene plotted and the *Z* scores of the individual highlighted as a dot.

We tested the impact of sex stratification on our multi-tissue outlier detection using GTEx data. We observed that males appeared to carry slightly more outliers than females on the X chromosome driven by a significantly higher number of under-expression outliers (Fisher’s exact test *p* = 0.01), with no significant difference in the number of over-expression outliers (*p* = 1) (Figures 2A and S7). On the autosomes there were no significant differences in the number of outliers between males and females. Further, despite equal sample size, there were fewer outliers in the male (*p* = 0.001) and female (p = 6e−05) groups on the autosomes compared with outliers in both groups, largely driven by significantly fewer over-expression outliers on the autosomes in the female (*p* = 7e−05) and male (*p* = 0.006) groups compared with both groups. As over-expression outliers are less likely to be driven by rare variants, this indicates that stratification can improve detection of genetically driven outliers. We were underpowered to analyze the impact of X chromosome inactivation and the pseudoautosomal regions (Figure S7C).^47^^,^^48^

We investigated genes detected as an outlier for an individual in only the sex-stratified or sex-combined group. The vast majority of genes maintain consistent z-scores regardless of group. We observed only 0.1% (27) X chromosome and 0.2% (951) autosomal gene-individual pairs change outlier status between sex-combined and sex-stratified groups (Figure 2B and S7; Table S1). Gene-individual pairs, consistently outliers in both the combined-sex and sex-stratified groups, showed an average *Z* score shift of 0.4 on the X and autosomes, and were mostly over-expression outliers. These genes were linked to 331 unique traits in OpenTargets and 4,062 unique traits in the GWAS catalog.^49^ For the gene *FAM9C*, one female is an outlier in the female-only group (*Z* = 4.1) but not in the combined group (*Z* = 2.0). *FAM9C* evolved independently on the X after recombination stopped with the Y, and often escapes XCI.^50^ For the gene *IL1RAPL2*, one male was an outlier (*Z* = 2.7) in the combined group but not in the sex-stratified group (*Z* = −0.5); this gene is in a region associated with X-linked nonsyndromic cognitive disability.^51^^,^^52^ Another gene, *G6PC2*, is an outlier for the female (*Z* = 7.4) and combined (*Z* = 3.9) groups. This gene is associated with release of glucose into the bloodstream and has been shown to have sex-specific effects in mice and chickens.^53^^,^^54^^,^^55^ There were more gene-individual pairs that were outliers in sex-combined but not the sex-stratified group (662 autosomes, 22 X chromosome) than the reverse, in which a gene-individual pair was an outlier in the sex-stratified but not the sex-combined group (289 autosomes, 5 X chromosome). We observed no genes with an outlier status change in the sign, further supporting that improvements in outlier detection due to sex stratification are due to uncovering previously masked effects.

**Figure 3:**
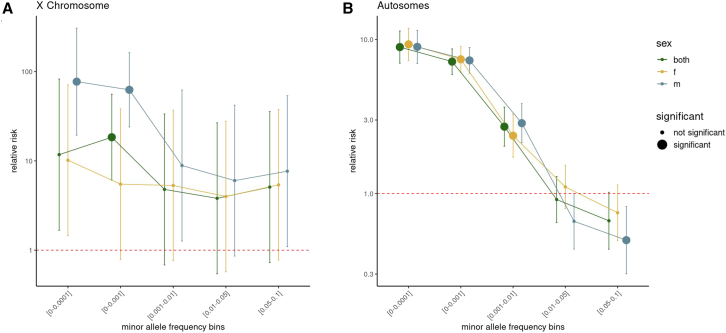
Impact of sex stratification on relative risks The relative risk of multi-tissue outliers having a nearby variant, with the additional line in a different color representing the both, male, and female groups for (A), the X chromosome and (B) the autosomes across minor allele frequency bins. This is plotted on a log-scale, and the point size indicates statistical significance. Error bars represent the 95% confidence intervals and *p* values are caculated using a Fisher's exact test and adjusted using BH.

Given that sex stratification impacts outlier detection, we investigated its impact on the enrichment of the proportion of outliers with a nearby variant compared with non-outliers. Across the autosomes, we observed no significant differences in enrichment (Figure 3). We have shown previously that outliers are more enriched for having nearby deleterious variants (higher CADD score).^16^ Males were enriched for rare deleterious (CADD > 15) variants on the X chromosome (Figure 3 and S8). This enrichment was driven by under-expression outliers (Figure S9). The point estimate for males was higher than females (males = 62.4, females = 5.5; Figure S8). In the male group, we observed a larger enrichment on the X chromosome for very rare variants compared with the autosomes (Figure S8).

### Discovery of sex-specific functional rare variants

Given the prevalence of sex-biased gene expression in human tissues, we hypothesized that rare variants could also manifest sex-specific effects.^5^ To test this, we trained a hierarchical Bayesian model, RIVER, using multi-tissue expression data from both sexes. RIVER significantly outperformed genomic annotation models in predicting outlier status of individuals with shared rare variants (MAF < 0.01) (Figure S10).^22^ We then applied this model to estimate the posterior probability for variants driving outlier status in female and male individuals separately. Overall, we scored 1.9 million rare variants on autosomes and 29 thousand variants on chromosome X. Sex-biased variants were defined to be those with a difference in the posterior probability more than 0.2.

**Figure 4:**
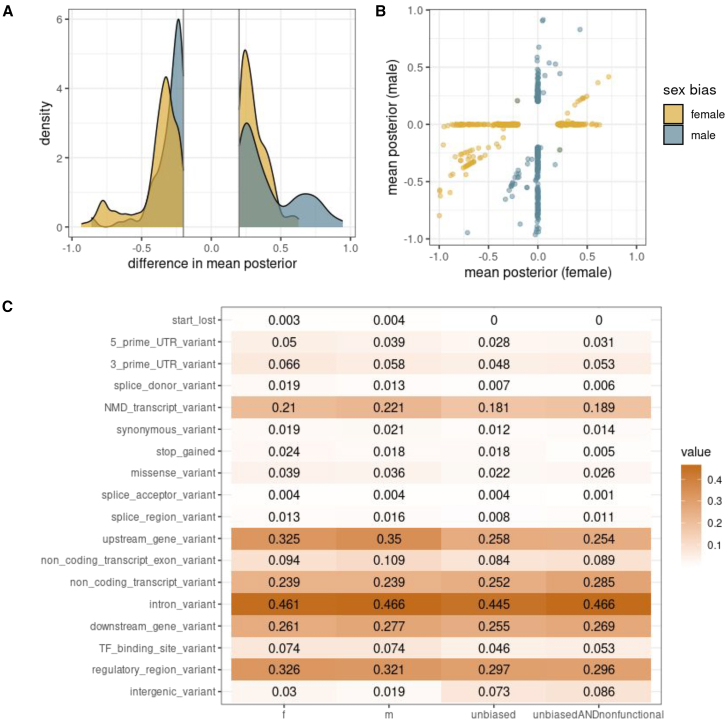
Sex-biased functional rare variants For predicted functional (abs(beta) > 0.2) sex-biased (absolute mean difference > 0.2) rare (MAF < 0.01) variants the (A) distribution of difference in mean posteriors between males and females, and (B) the values of the posteriors in each sex. (C) For each given variant category, the proportion of rare variants that had this annotation. Note that variants can be in multiple categories. This is calculated for female-biased, male-biased, unbiased, and unbiased and also non-functional rare variants.

The vast majority of variants shared similar functional predictions in both sexes (99.5% with posterior probability difference <0.1**)**; however, we identified 5,245 variants with sex-biased posteriors. For increased confidence, we then only included variants that were sex biased in at least 1 of 5 permutations of the sex labels, reducing our set to 733 variants across 450 genes on the autosomes, and 10 variants across 8 genes on the X chromosome (Table S2; Figure 4A, see methods). There were a similar number of female-biased and male-biased functional rare variants (Table S3). There was no ontological enrichment for genes with a sex-biased variant.^56^ On the X chromosome, there was a variant (chrX:124223332) predicted to increase expression only in males of the gene *SH2D1A*. This gene has been linked to X-linked lymphoproliferative disease, which causes the immune system to overreact primarily in males in response to Epstein-Barr virus.^57^

To investigate the functional impact of sex-specific rare variants, we curated a comprehensive set of annotations. We then examined the proportion of functional rare variants (defined as RIVER posterior >0.2) with a given annotation (missense, intronic, etc.) and did so for those that were female biased, male biased, and not sex biased. Here, we defined the background as all scored variants (appearing in both females and males), and sex-shared variants as those with large posteriors (>0.2) in both sexes. Several variant types were enriched for being sex-biased and functional compared with non-functional, such as TF binding sites, stop-gained, and splice sites (Figures 4B, 11, and 11B). Non-functional variants were enriched in non-coding and intergenic regions (Figures 4B, 11A, and 11B).

There was a significant overlap of 11 sex-biased eQTLs genes from Oliva et al. and our sex-biased rare variant list (hypergeometric test *p* = 0.008), and a significant overlap of 37 sex-biased eQTL genes from Jones et al. (hypergeometric test *p* = 5.7e−6; Figure S12A).^5^^,^^58^ The gene *TDRD12*, which has been linked to fertility, overlapped in all three sets.^59^^,^^60^ When considering tissue-specific scores from Oliva et al. the adrenal gland, which has high hormonal activity and sexual dimorphism, had the largest correlation to the sex-biased rare variants (Figures S12B; Table S4).^61^

We identified two rare variants in two genes that were predicted to exhibit sexual antagonism (absolute mean posterior greater than 0.2 in both sexes with different directions). The variant chr11:75430623 was predicted to decrease expression of *KLHL35* in females and increase expression in males (Figure S12C). Increased expression of *KLHL35* has been linked to survival levels for lung adenocarcinoma with a *TP53* mutation,^62^ and females have been shown to have better survival of this disease.^63^ The stop-gain variant chr1:171636338 was predicted to increase expression in females and decrease expression in males for the gene *MYOC*, and had a CADD score of 37 and is likely pathogenic for glaucoma (Figure S12D). Mutations in this gene can cause glaucoma, and variants in other glaucoma-causal genes have been linked to sex bias of this disorder (see Suri et al.^63^). *MYOC* has also been linked to sex-determination in zebrafish.^64^

### Sex-specific functional rare variants occur in genes with known sex-biased drug interactions

Rare variants account for an estimated 30%–63% of variation in drug response,^65^^,^^66^^,^^67^ and approximately half of known pharmacogenes are exclusively driven by rare variants.^68^ It has further been observed that 77%–96% of individuals contain at least one deleterious rare variant in an actionable pharmacogenetic gene.^69^^,^^70^^,^^71^^,^^72^ We sought to determine if the role of predicted functional sex-biased variants were relevant to pharmacogenetics. To do so, we compared 743 functional sex-biased rare variants (from 458 genes) with 5,040 functional non-sex-biased rare variants (from 2,516 genes) across pharmacogenetic databases.

**Figure 5:**
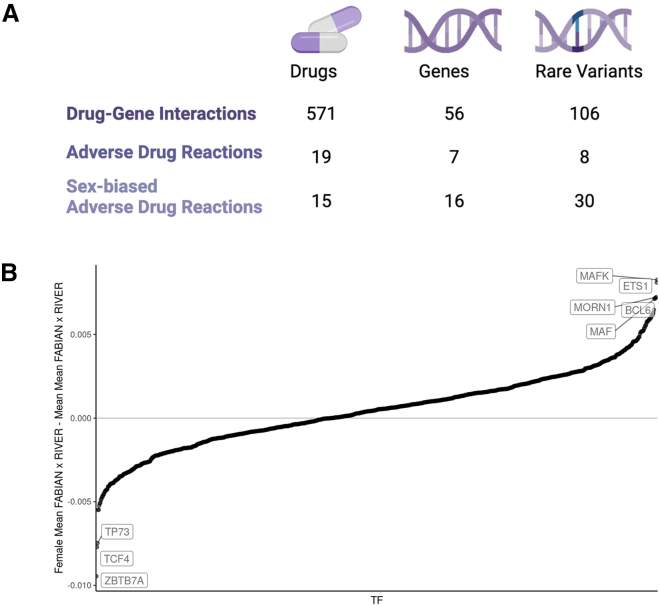
Sex-biased functional rare variants (A) Across the DGIdb, the FDA list adverse drug reactions, and adverse drug reactions with known sex biases from Zucker and Prendergast,^38^ the number of drugs and genes that were linked to a sex-biased rare variant. (B) For each transcription factor, ordered with positive values representing increased binding in females compared with males, and negative being the reverse, the gene-summarized difference in the mean female FABIAN scores × RIVER scores from the mean male scores.

To connect functional rare variants to genes that are of pharmacogenetic interest we used the DGIdb, which contains 13,186 drugs with known 6,888 gene interactions.^36^ We identified 106 sex-biased functional rare variants from 56 unique genes that interact with 571 unique drugs (Figure 5A and S13A; Table S5). One such variant was the intronic variant chr16:28607405, which was predicted to increase expression of *SULT1A1* only in females. This gene plays a known role in estrogen metabolism.^73^^,^^74^ Of these unique drugs, 491 interacted with a gene that had a female-biased functional rare variant, while just 177 interacted with a gene that had a male-biased functional rare variant (Figures S13A; Table S5).

It has been observed that adverse drug reactions occur at a higher rate in women,^75^ and there are over 20,000 predicted adverse drug effects with sex differences.^76^ The FDA lists 131 drugs with adverse drug reactions that have been linked to a gene.^37^ We identified 8 functional sex-biased rare variants from 7 genes linked to 19 drugs with adverse drug reactions (Figure 5A). Despite having a similar number of functional sex-biased rare variants (5 female and 3 male), females had more associated drugs with these adverse reactions (17 female, 2 male) (Figure S13A; Table S5). Of 59 drugs previously identified to have sex differences in adverse drug reactions, 15 were linked to a gene which also had a sex-biased functional rare variant (Figures 5A and S13A; Table S5).^38^ This was enriched compared with drugs linked to genes with non-biased functional rare variants (relative risk = 1.67, *p* = 0.005) (Table S6).

One of the largest number of functional rare variants with a predicted sex bias linked to a single drug was 13 variants across 4 genes linked to doxorubicin, which has known sex differences in toxicity.^77^ We identified four functional rare variants in the gene *CBR1*, all of which were male biased and predicted to reduce expression (Figure S13B). *CBR1* is a reductase of doxorubicin, and upregulation of this gene drives doxorubicin resistance,^78^ which leads to increased myotoxicity, of which there are known sex differences for this drug.^79^ Therefore, reduced expression of this gene is preferred, and reduction of *CBR1* in mice improved doxorubicin efficacy only in male mice.^80^ We also identified five functional rare variants predicted to decrease expression of the gene *NDUFS3* only in females, and one functional rare variant predicted to increase expression of *NDUFAF3* only in males. Metformin inhibits both of these genes; females are known to need a lower dose and have more adverse drug reactions than males.^36^^,^^81^

### Identification of sex-specific TF networks

Given that TF activity has been linked to sex-biased gene expression across human tissues, we hypothesized that sex-biased variants in regulatory regions may inform discovery of TFs and TF networks with sex biases.^82^ To this end, we first performed a motif enrichment analysis using HOMER with a 100 bp window on both sides of female- and male-biased rare variants on the autosomes.^39^ The top known motif was a target of the gene *SIX1* (Figure S14A). *SIX1* is modulated via a microRNA intermediated by the highly sex-biased gene *XIST*, *SIX1* can modulate a key regulator of the sex determination pathway *SRY*, and *SIX1* knockout reduced male gonadal development in mice.^83^^,^^84^ The top *de novo* enriched motif was a target of *HAND2* (Figure S14B). This gene plays a large role in pregnancy and the menstrual cycle, is a downstream target of estrogen and progesterone signaling, and can also regulate estrogen.^85^^,^^86^

We then use FABIAN variant, an *in silico* modeling approach based on a large collection of TF motif databases that identifies TFs likely disrupted by variants, to further understand the impact of the sex-biased rare variants on TFs.^40^ We multiplied the FABIAN-variant score with the RIVER score, and signed it such that positive meant potential gain of binding and negative meant potential loss of binding. With this, we were able to identify TFs for target genes whose motifs were predicted to be disrupted by sex-biased variants (Figure S15A). For example, *YY1*—a TF known to regulate sex-biased transcription—was identified as a TF targeting a gene with predicted sex-biased rare variants (Figure S15A).^87^ We further identified TFs with a difference in gain or loss of binding for sex-biased rare variants (Table S7). For example, *BCL6* was identified as predicted increased binding in females but decreased binding in males (Figures 5A and S13B; Table S7). *BCL6* has been shown to preferentially bind to female-biased targets.^88^ Overall, our data suggest that rare variants can mediate sex-specific regulatory functions through altered binding of sex-biased TFs.

## Discussion

Previous studies have examined the impact of rare variants across the autosomes using outlier-based approaches.^17^^,^^18^^,^^19^^,^^20^^,^^21^ However, to date, none of these approaches have been applied to the X chromosome. In this work, we extended this approach to identify multi-tissue outliers on the X chromosome, and found that these outliers were enriched for nearby rare variants.

We demonstrated that rare variants are more enriched for gene expression outliers on the X chromosome in males compared with females. This follows the intuition that the additional copy of the X chromosome in females may compensate for some extreme variant effects, and follows patterns of X-linked mutations having stronger impacts in males.^89^ We also observed significantly fewer over-expression outliers on the autosomes in the sex-stratified groups compared with the sex-combined group, indicating that this approach removes some false outliers. This emphasizes the importance of X chromosome and sex-stratified analyses particularly for genes with large differences in mean or variance between sexes.

Sex-biased eQTL studies have demonstrated the power challenges with uncovering genetic effects. However, we reasoned that machine learning approaches that combined molecular outliers and variant annotation, when trained on sex-stratified datasets, may elucidate candidate causal variants. Using this approach, we identified 753 rare variants with different predicted effects between males and females, and observed that these sex-biased rare variants are enriched in specific regulatory regions and annotations. We identified just two genes, *KLHL35* and *MYOC*, that had rare variants with predicted effects in opposite directions between males and females. This supports the hypothesis by Zhu et al. that genetic variants modify sex differences primarily through amplification, in which sex differences are modulated by magnitude, rather than through direction of effect.^90^ As machine learning models predicting variant effects evolve, we highlight the advantages to considering sex as an important variable, not just to regress out, but to stratify in training and testing. For example, in an Alzheimer’s dataset, sex-stratified training led to better disease prediction and highlighted different underlying pathways.^91^ This indicates that sex-stratified model training can elucidate sex-specific variant effects, and demonstrate a strong impact for X chromosome variants.

Biological sex has been documented to influence pharmacokinetics, pharmacodynamics, adverse drug reactions, and toxicity, but there remain challenges identifying the biological mechanisms.^92^^,^^93^^,^^94^ Rare variants are also known to have a large impact on pharmacogenes.^68^ We identified a functional sex-biased rare variant in 0.8% of known pharmacogenes in DGIdb.^36^ Genes with adverse drug reactions were enriched for containing sex-biased functional rare variants, including in clinically actionable genes. This suggests that functional rare variants that act differently between males and females may contribute to sex differences in pharmacogenetics and adverse drug reactions. However, such candidates remain to be functionally evaluated.

One hypothesis for how sex-biased variants ultimately impact expression is through sex-biased expression of TFs. Jones et al. found that sex-biased eQTLs are predominantly targeted by TFs with sex differences.^58^ We found that sex-biased rare variants significantly overlapped with sex-biased common variants in their target genes. In this study, we were able to replicate sex-biased TFs using enrichment analysis of genomic regions defined by sex-specific rare variants. These results provide intriguing possible mechanisms of sex-biased TF regulatory networks across human tissues,^82^ in which both common and rare genetic variants contribute to sex-biased transcription profiles through modulation of TF binding in sex-specific manners.^58^ Importantly, permutation of sex labels failed to replicate these findings, suggesting that our sex-stratified RIVER models are robust in discovering sex-specific gene regulatory networks anchored by rare genetic variants. Screening for motif enrichment around sex-biased variants identified several targets of genes involved in highly sex-biased genes, and relevant TFs with known sex differences in binding, such as *BCL6*. Future analysis on the differential effects of sex-specific rare variants on complex traits could further elucidate the specific TFs and relevant tissues underlying observed sex differences.

There are several limitations to consider. Our analyses refer to XY males and XX females, and exclude intersex individuals or individuals with other combinations of sex chromosomes due to a lack of power and as such do not capture the full spectrum of individuals. A further limitation is that the GTEx population is primarily European, and we have filtered to only Europeans given underlying gaps in reference data that would misclassify rare variants. Understanding rare variants in a diverse dataset is both a scientific and ethical priority.^95^^,^^96^ We were not powered enough to do analyses within the X chromosome such as investigating the impact of X chromosome inactivation (and subsequent escape), and a comparison between the pseudoautosomal and non-pseudoautosomal regions. There are opportunities for future papers to explore how these patterns vary for indels and structural variants. While sex differences are important to understand, as many diseases vary in risk and severity by sex (see Khramtsova et al.^4^), it is also important to not over-report sex differences.^97^ Overall, males and females were very similar in our analyses; reflecting Lewontin’s observations for ancestry that, when it comes to genetic effects, there are more within-sex differences than between-sex differences.^5^^,^^98^

Genetics studies of molecular and trait phenotypes are increasingly elucidating the impacts of low-frequency and rare variants.^99^ However, for the foreseeable future many rare and ultra-rare variants will be difficult to interpret using conventional association testing. In this study, we survey the impact of rare variants on gene expression on the X chromosome, note how they differ by sex, and implicate further pharmacogenetic and TF mechanisms that can create sex differences.

## Data and code availability

Scripts used for analysis are available at GitHub (github.com/raungar/rarevariant_x_sex). Data are available to authorized users through dbGaP (phs000424.v8) and on the GTEx portal.

## Acknowledgments

Thank you to the donors and family of donors from the GTEx study for making this research possible. This work utilized computing resources provided by the Stanford Genetics Bioinformatics Service Center, supported by NIH Instrumentation Grant S10 OD025082, and would not have been possible without the support of the Stanford SCG cluster system administrators. We thank the Montgomery lab members and Stanford Genetics department members for comments and suggestions throughout the research process. Thank you to R.A.U.’s committee members Noah Rosenberg, Marcia Stefanick, and Jesse Engreitz for feedback over the years. R.A.U. was funded by the Stanford Genome Training Project (NIH T32HG000044) and GREGoR Consortium (NIH U01HG011762). S.B.M. was supported by NIH grants U01HG012069 and R01MH125244. The content is solely the responsibility of the author and does not necessarily represent the official views of the National Institutes of Health.

## Author contributions

R.A.U. and S.B.M. conceived the study. R.A.U., T.L., and S.B.M. significantly contributed to study design, with feedback from A.B. and N.E. Analyses were performed by R.A.U., T.L., and N.G.V. with mentorship from S.B.M. and A.B. Figures were generated by R.A.U. and the manuscript was primarily written by R.A.U. with major contributions from T.L. and edits provided by S.B.M. All authors provided feedback on the manuscript to improve it.

## Declaration of interests

During this project R.A.U. was employed for an internship by Vertex Pharmaceuticals. N.E. contributed to this project exclusively during her PhD. S.B.M. is an advisor to Character Bio, MyOme, PhiTech, and Tenaya Therapeutics. A.B. is a co-founder and equity holder of CellCipher, Inc., a stockholder in Alphabet, Inc., and has consulted for Third Rock Ventures.

## Web resources

GitHub, github.com/raungar/rarevariant_x_sex

GTEx portal, https://gtexportal.org/

## Supplemental information

Data S1. Table S1–S7

Document S2. Article plus supplemental information
