# Supplementary material for: Transcriptomic signatures of rare variant impacts across sex and the X chromosome
Source: HGG Adv. 2025 May 31;6(3):100463. doi: 10.1016/j.xhgg.2025.100463 (PMC12214130; doi:10.1016/j.xhgg.2025.100463)
Supplement: Document S1. Figures S1–S15 [file mmc1.pdf]

HGGA, Volume *6*

Supplemental information

Transcriptomic signatures of rare variant impacts across sex and the X chromosome

Rachel A. Ungar, Taibo Li, Nikolai G. Vetr, Nicole Ersaro, Alexis Battle, and Stephen B. Montgomery

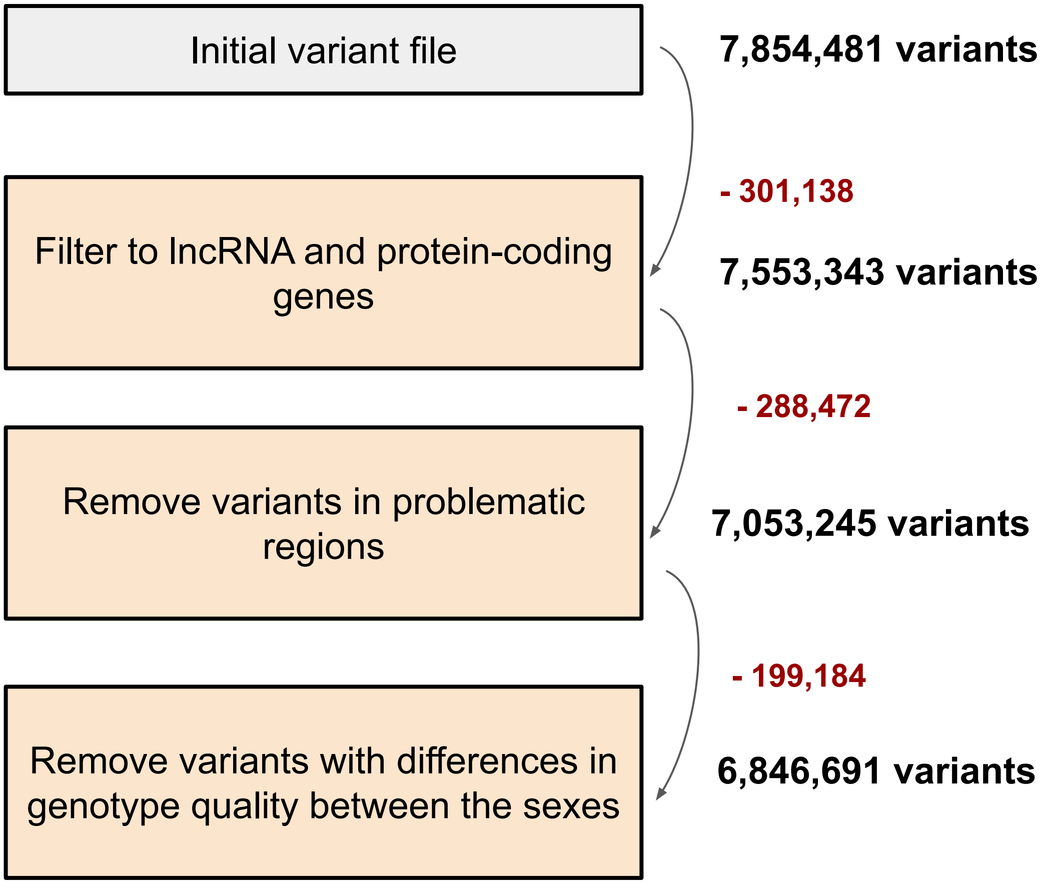

Supplemental Figure 1: Variant filtration. Variant filtration process. In red is the number of variants removed for each after each filtration process.

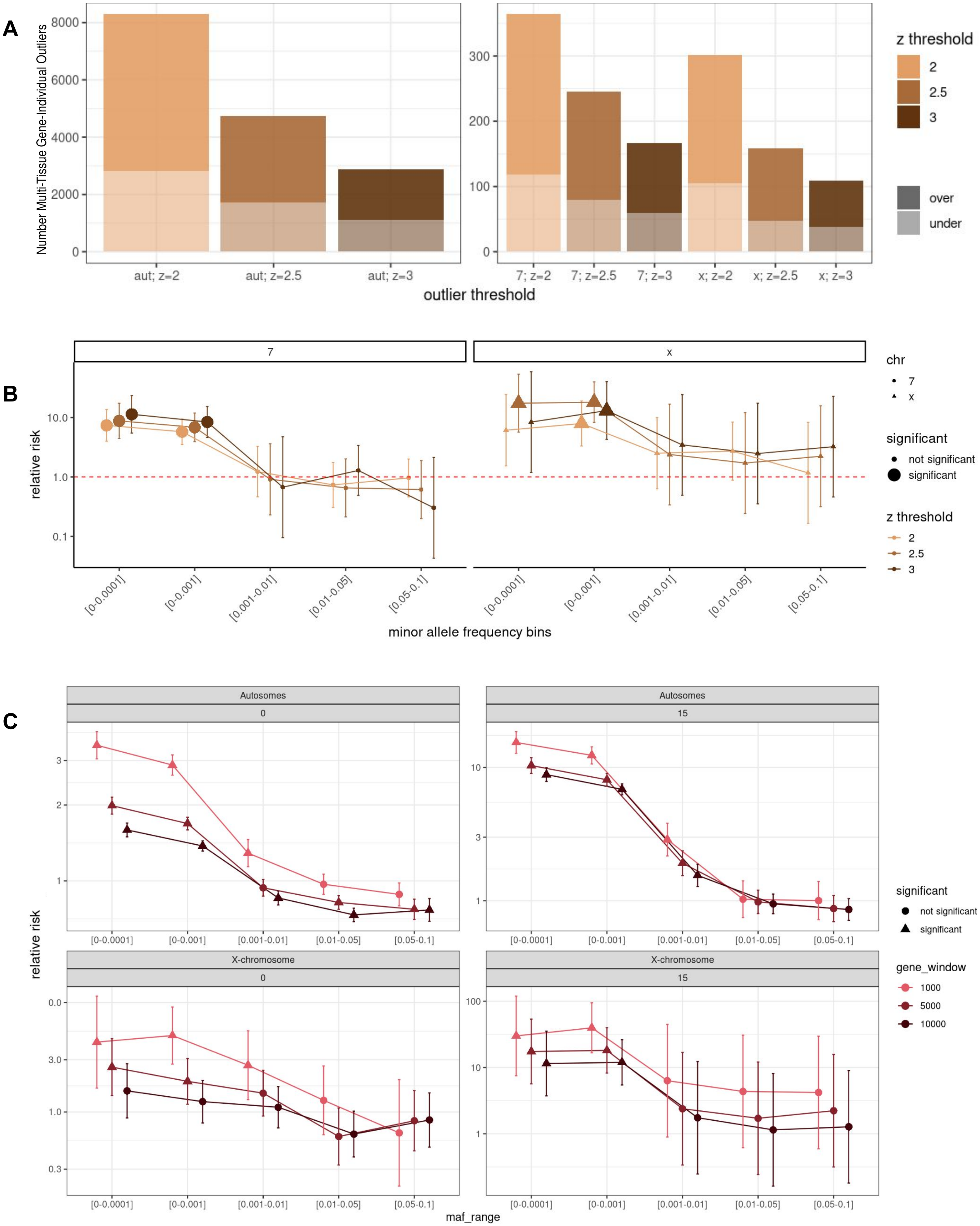
A
Number Multi-Tissue Gene-Individual Outliers
8000
6000
4000
2000
0
300
200
100
0
aut; z=2
aut; z=2.5
aut; z=3
7; z=2
7; z=2.5
7; z=3
x; z=2
x; z=2.5
x; z=3
outlier threshold
z threshold
2
2.5
3
over
under
B
7
x
relative risk
10.0
1.0
0.1
[0-0.0001]
[0-0.001]
[0.001-0.01]
[0.01-0.05]
[0.05-0.1]
minor allele frequency bins
chr
7
x
significant
not significant
significant
z threshold
2
2.5
3
C
Autosomes
0
Autosomes
15
X-chromosome
0
X-chromosome
15
relative risk
3
2
1
10
3
1
0.0
3.0
1.0
0.3
100
10
1
[0-0.0001]
[0-0.001]
[0.001-0.01]
[0.01-0.05]
[0.05-0.1]
maf_range
significant
not significant
significant
gene_window
1000
5000
10000

Supplemental Figure 2: Impact of parameterizations. Data here includes all sexes. **a**, The number of multi-tissue gene-individual outliers that occur at different z-score thresholds across the X chromosome, and chromosome 7. **b**, The impact of z-score thresholds on relative risk enrichments for the autosomes and X chromosome. **c**, Impact on relative risk enrichments of the gene window which specifies how far away variants are to a given gene. This is done across the X chromosome and autosomes, as well as a CADD threshold of zero and fifteen. For b and c, error bars represent the 95% confidence interval, and the p-value was calculated using a Fisher's exact test and adjusted using Benjamini-Hochberg.

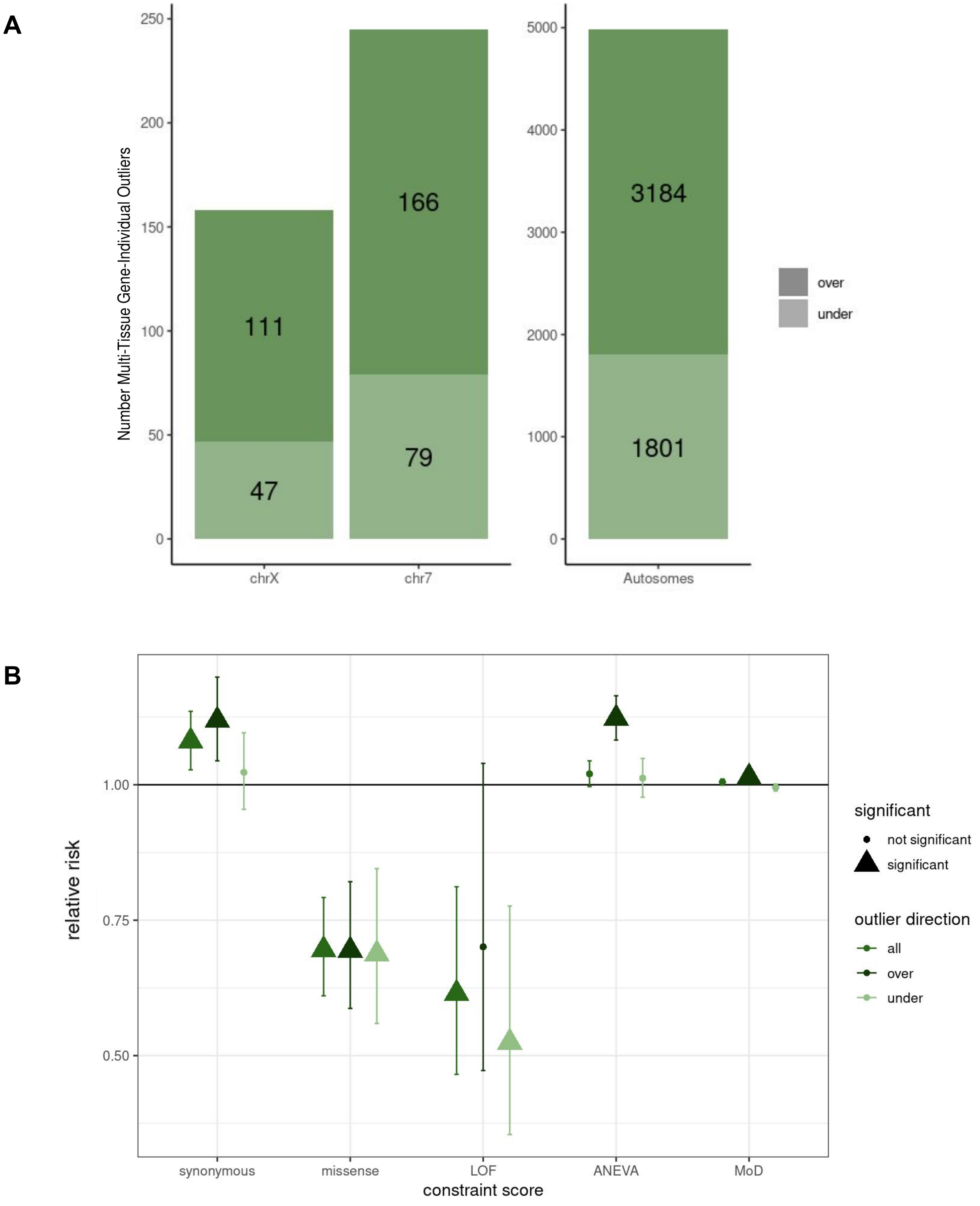

Supplemental Figure 3: Over and under-expression outlier summary. a, Number of multi-tissue gene-individual outliers across all individuals (male and female) split by over/under where the lighter shade is under-expression outliers, and the darker shade is over-expression outliers. **b,** Relative risk of outliers having a high (synonymous/missense/LOF from Samocha et al. z-score >2, ANEVA from Mohammadi et al >0.95, MoD from Dong et al > .95) constraint score as compared to a low score (synonymous/missense/LOF from Samocha et al. z-score < -2, ANEVA from Mohammadi et al <.05, MoD from Dong et al <.05)). Significance is Benjamini-Hochberg corrected and calculated with a Fisher's exact test, and error bars represent the 95% confidence intervals. This is done for all outliers, over-expression outliers, and under-expression outliers. Significant enrichments are a large size and represented with a triangle.

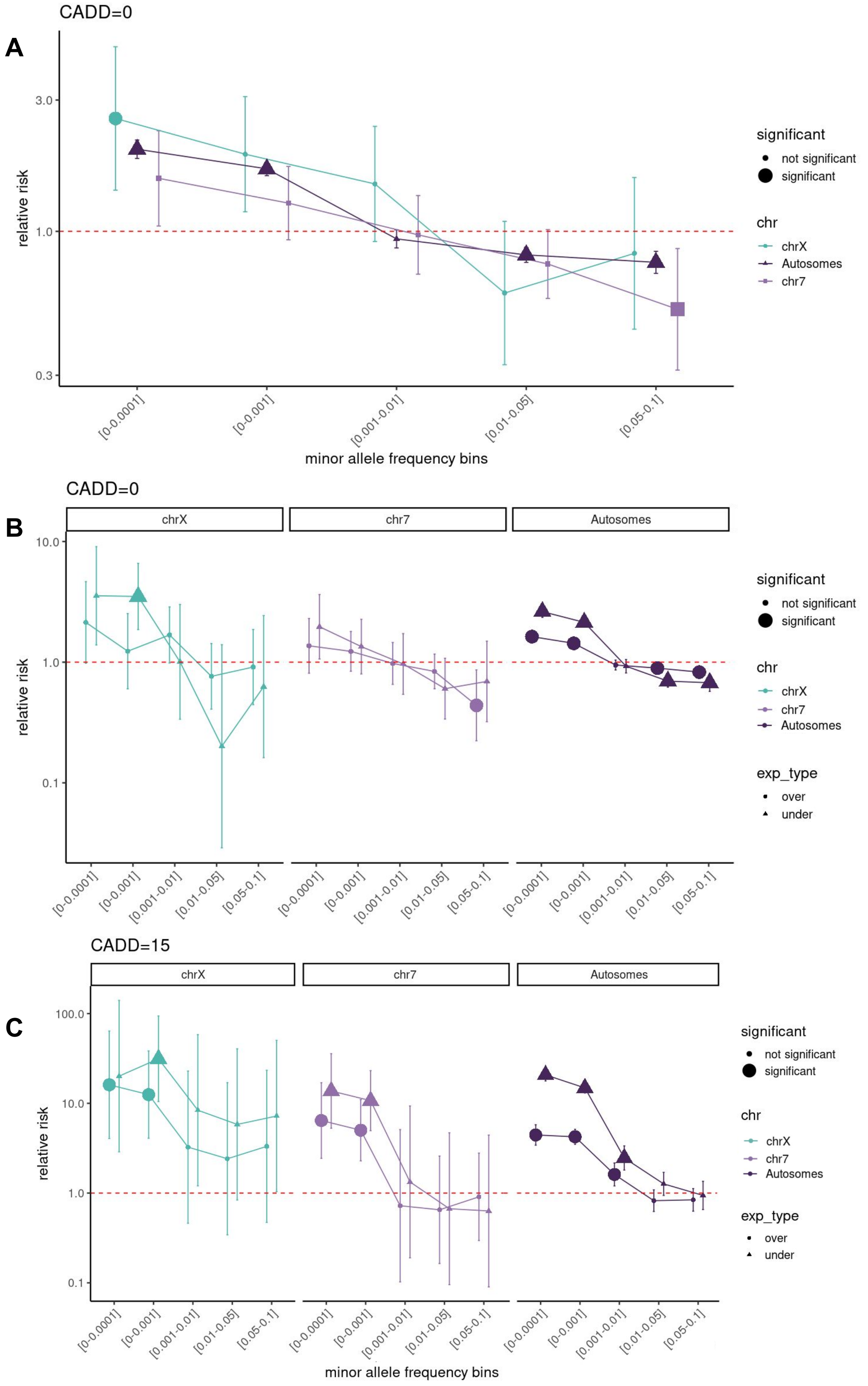

Supplemental Figure 4: Relative risk enrichments. The relative risk of having a multi-tissue outlier with a nearby variant within a given frequency bin as compared to a non-outlier. Error bars represent the 95% confidence interval, and the p-value was calculated using a Fisher's exact test and adjusted using Benjamini-Hochberg. In **a,** this is done with no CADD threshold and is colored by chromosome. Enrichments were calculated for under-expression and over-expression outliers across the X chromosome, chromosome 7, and the autosomes at **b,** no CADD threshold and **c,** a CADD threshold of 15. Point size represent a significant enrichment, and in b, and c, circles represent over-expression outliers, and triangles represent under-expression outliers.

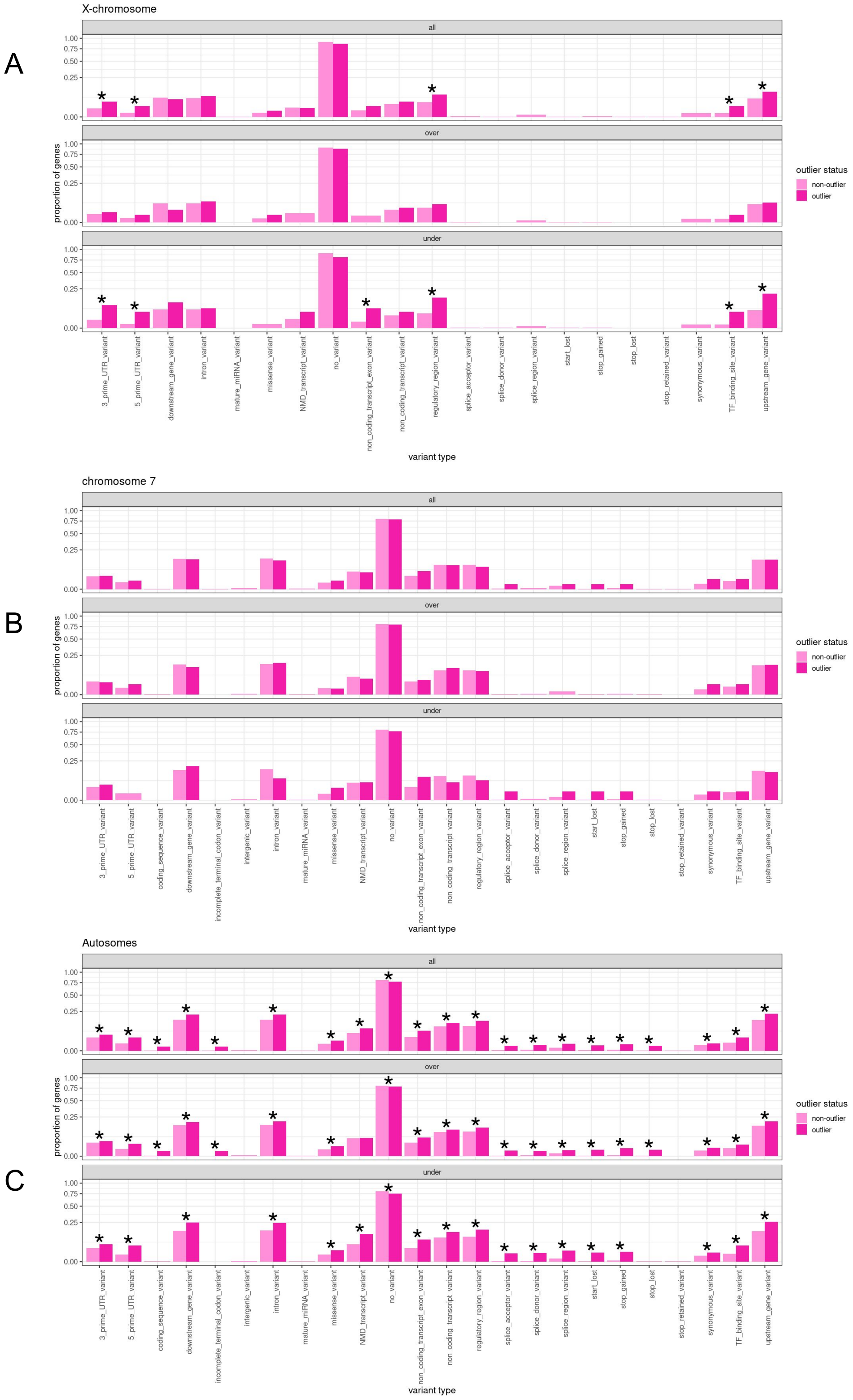

Supplemental Figure 5: Enrichment of variant types. The proportion of outliers (bright pink) with a given rare variant (MAF<0.01) as compared to the non-outliers (light pink) with a given variant across **a,** the X chromosome, **b,** chromosome 7**,** and **c,** the autosomes. Significance level is determined by Fisher's exact test and adjusted using the Benjamini-Hochberg approach for multiple testing correction and is denoted with an asterisk.

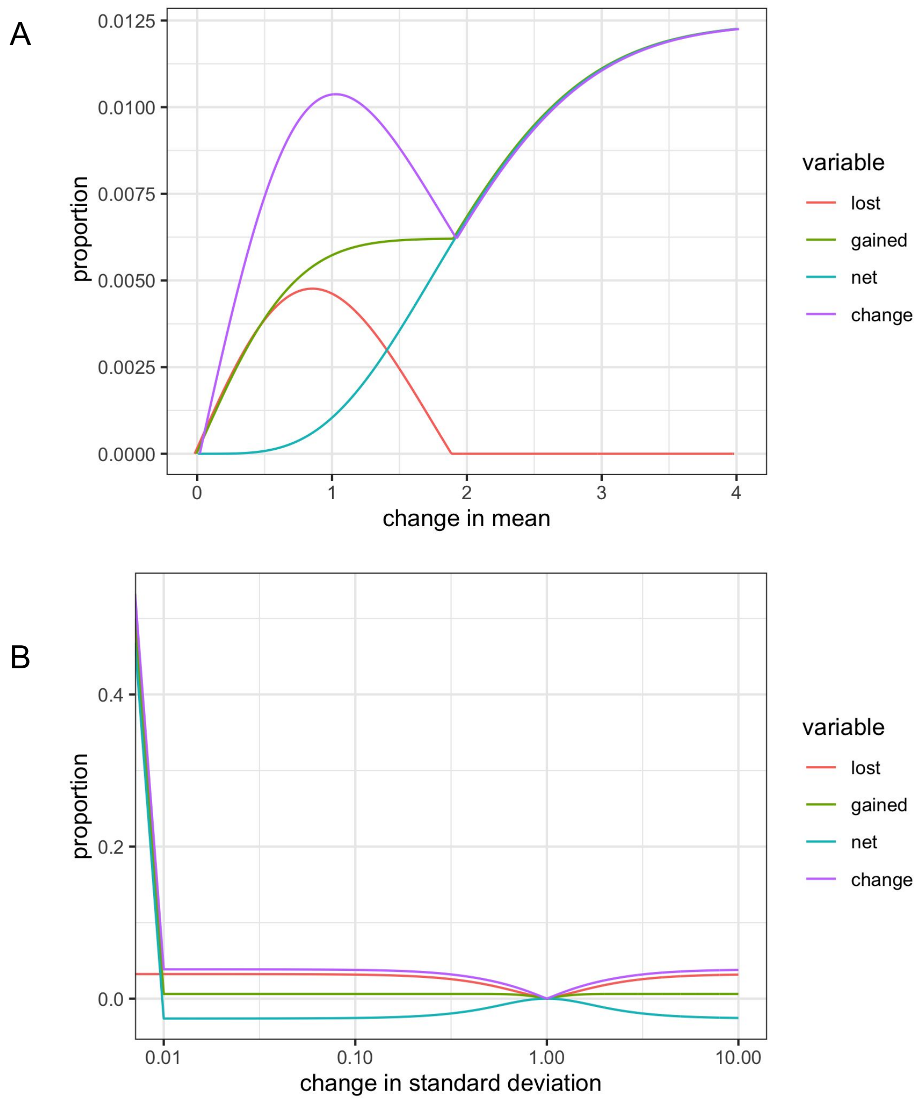

Supplemental Figure 6: Theoretical impact of sex-stratification. The impact of changing the **a,** mean or **b,** standard deviation on the proportion of genes that will either lose outlier status (red) and gain outlier status (green). The change (purple) represents the sum of the positive and negative change, while the net change (blue) represents the sum of the absolute value of positive and absolute value of negative change.

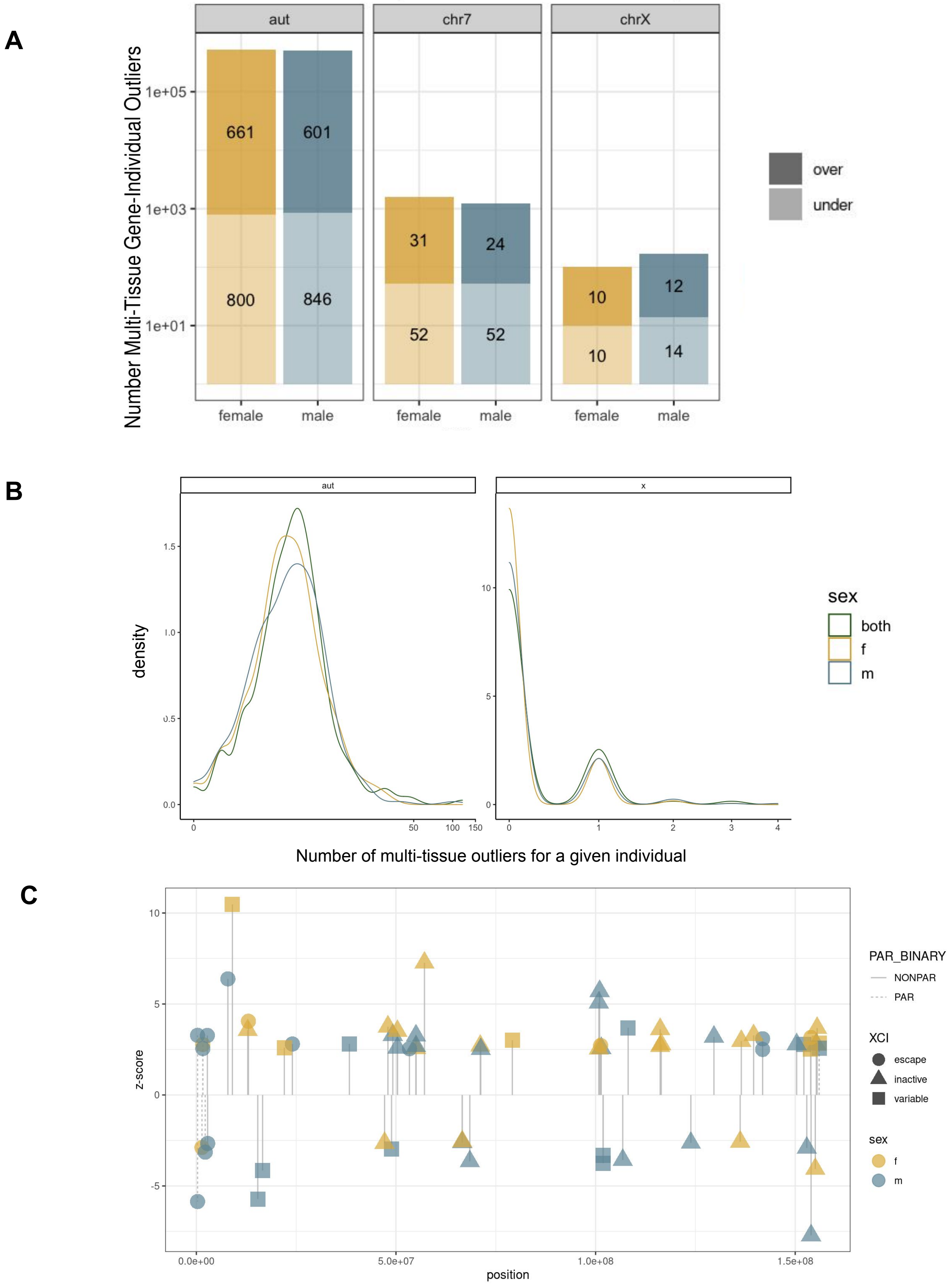

Supplemental Figure 7: Number of outliers sex-stratified. a, The number of multi-tissue gene-individual outliers split by over (darker, on top) and under (lighter, on bottom), across the autosomes, X chromosome, and chromosome 7. **b,** the distribution of the number of outliers per individual stratified by sex for the autosomes and X chromosome. **c,** Across the X chromosome the multi-tissue gene-individual z-score is plotted for outliers. The line type represents if it's in the PAR (dashed) or non-PAR (full) region defined by Webster et al. A circle represents if the gene escapes X chromosome inactivation, the triangle represents genes that never escape X-inactivation, and the square represents genes that sometimes escape X-inactivation using data from Tukiainen et al.

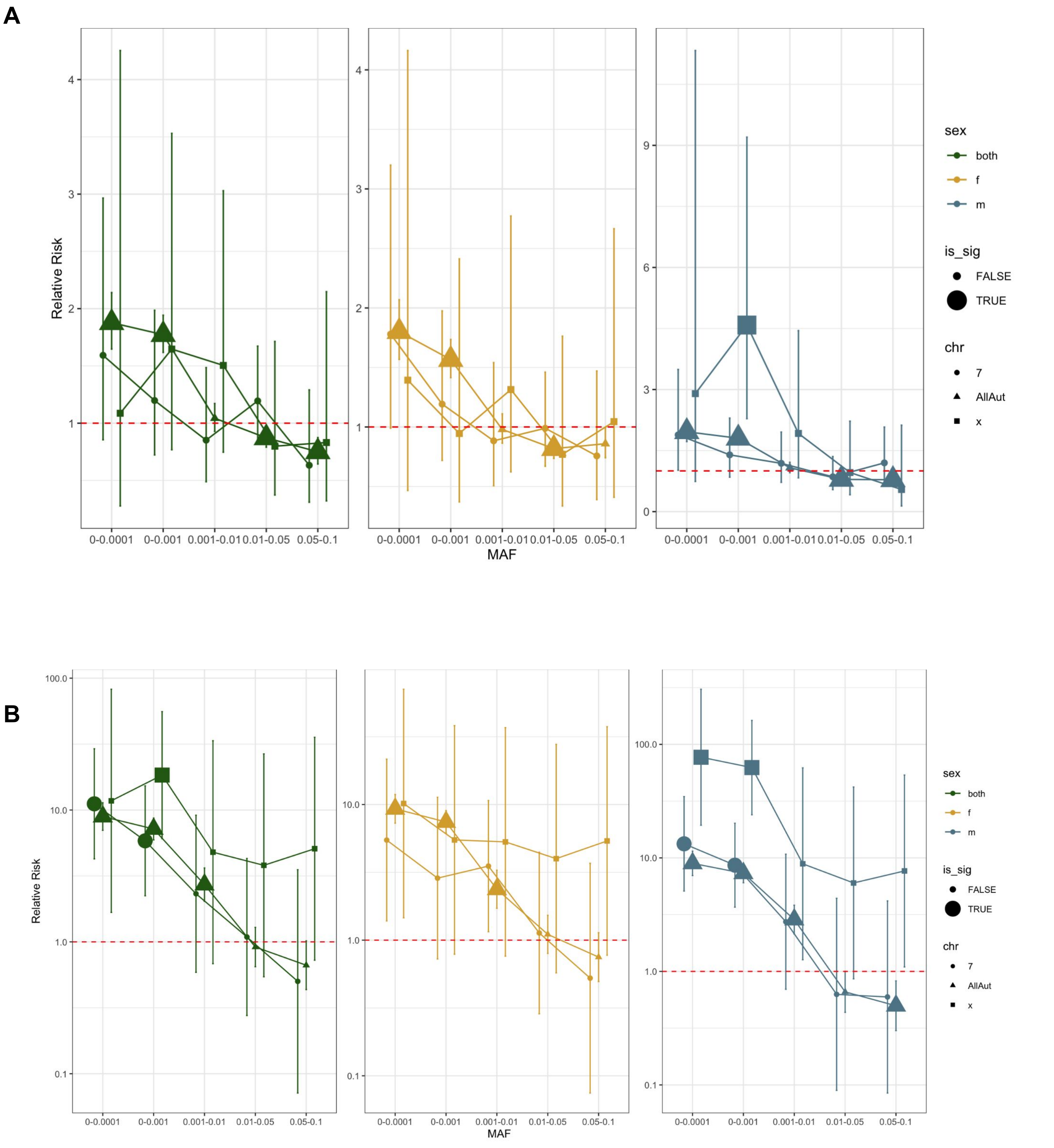

Supplemental Figure 8: Sex-stratified enrichment scores. Enrichment for multi-tissue outliers having nearby variants within a given frequency bin as compared to non-outliers at **a,** no CADD threshold and **b,** a CADD threshold of at least 15. This is across the X chromosome, chromosome 7, and autosomes and stratified by sex. Significance is size of dot. Shape represents the chromosome, and color represents sex. Error bars represent the 95% confidence interval, and the p-value was calculated using a Fisher's exact test and adjusted using Benjamini-Hochberg.

A

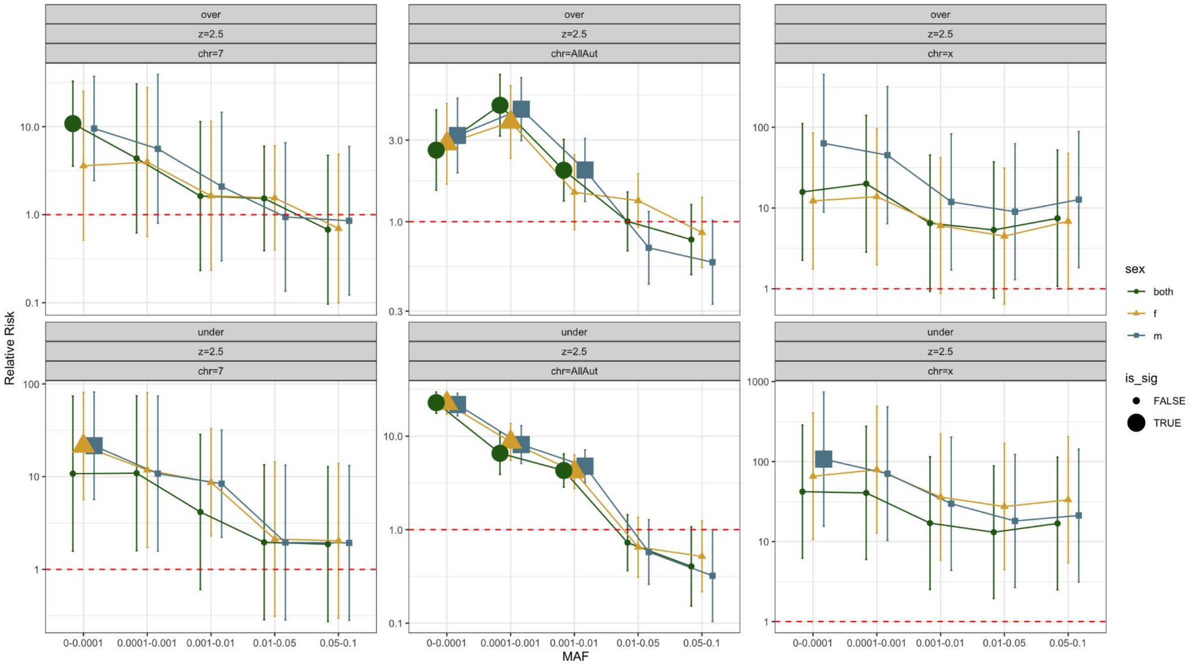

B

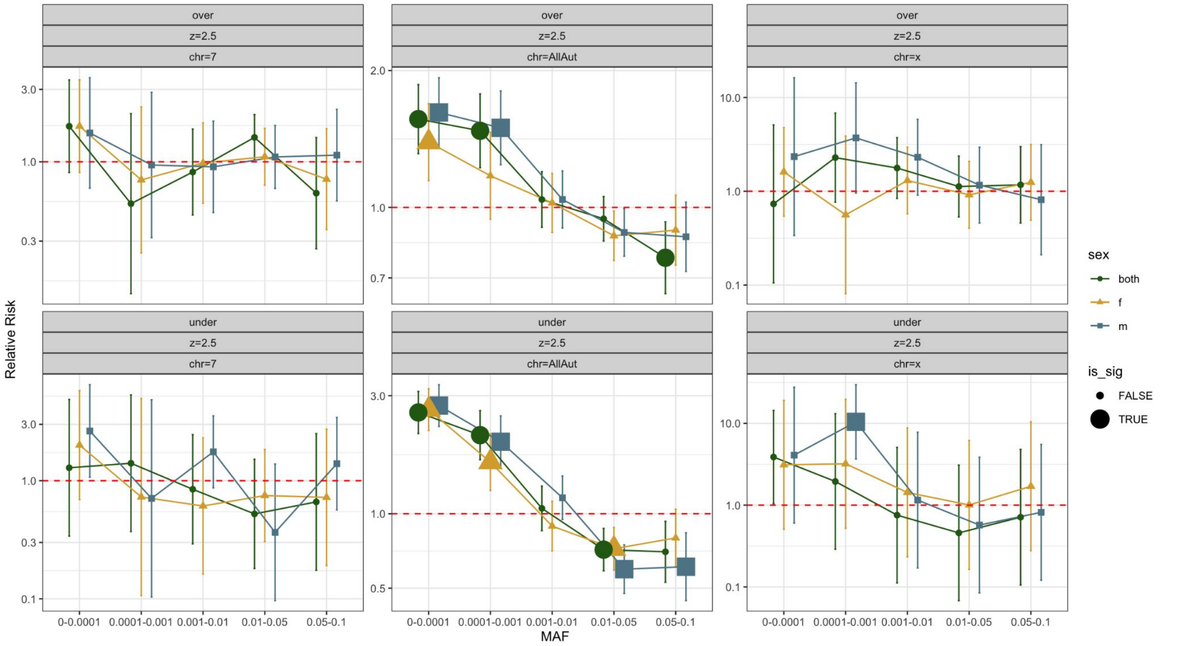

Supplemental Figure 9: Sex-stratified enrichment scores split by under- and over-expression. Enrichment for multi-tissue outliers having nearby variants within a given frequency bin as compared to non-outliers at **a,** no CADD threshold and **b,** a CADD threshold of at least 15. This is across the X chromosome, chromosome 7, and autosomes and stratified by sex. This is for both over- and under-expression outliers. Significance is size of dot, color represents sex. Error bars represent the 95% confidence interval, and the p-value was calculated using a Fisher's exact test and adjusted using Benjamini-Hochberg.

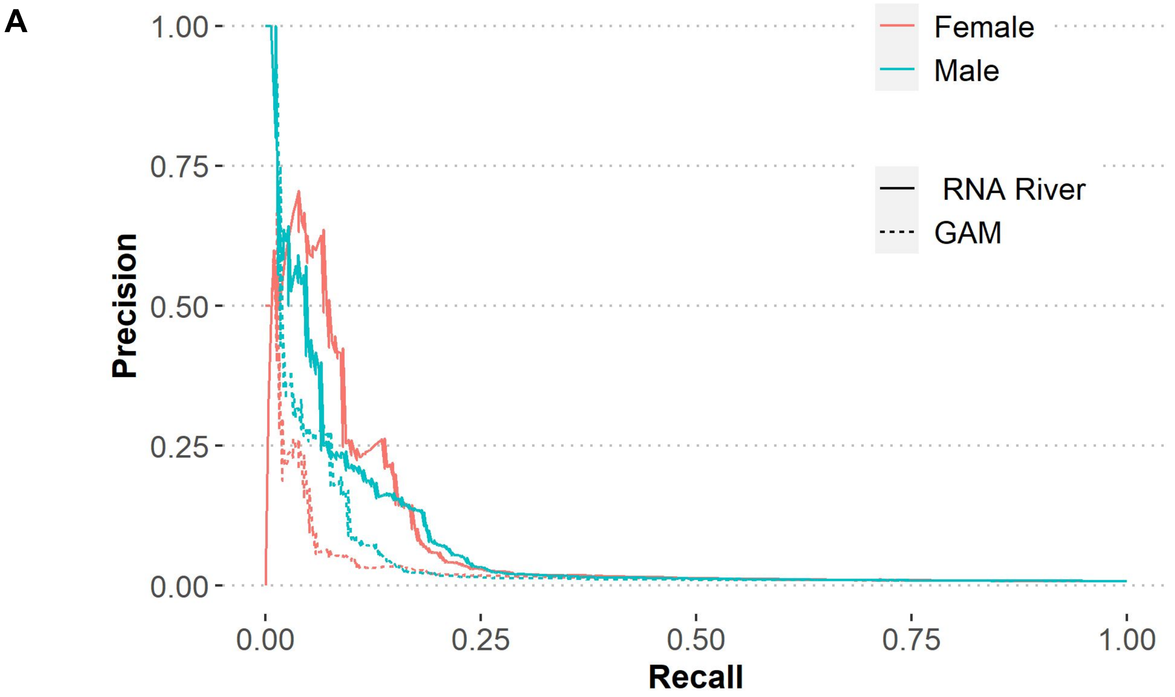

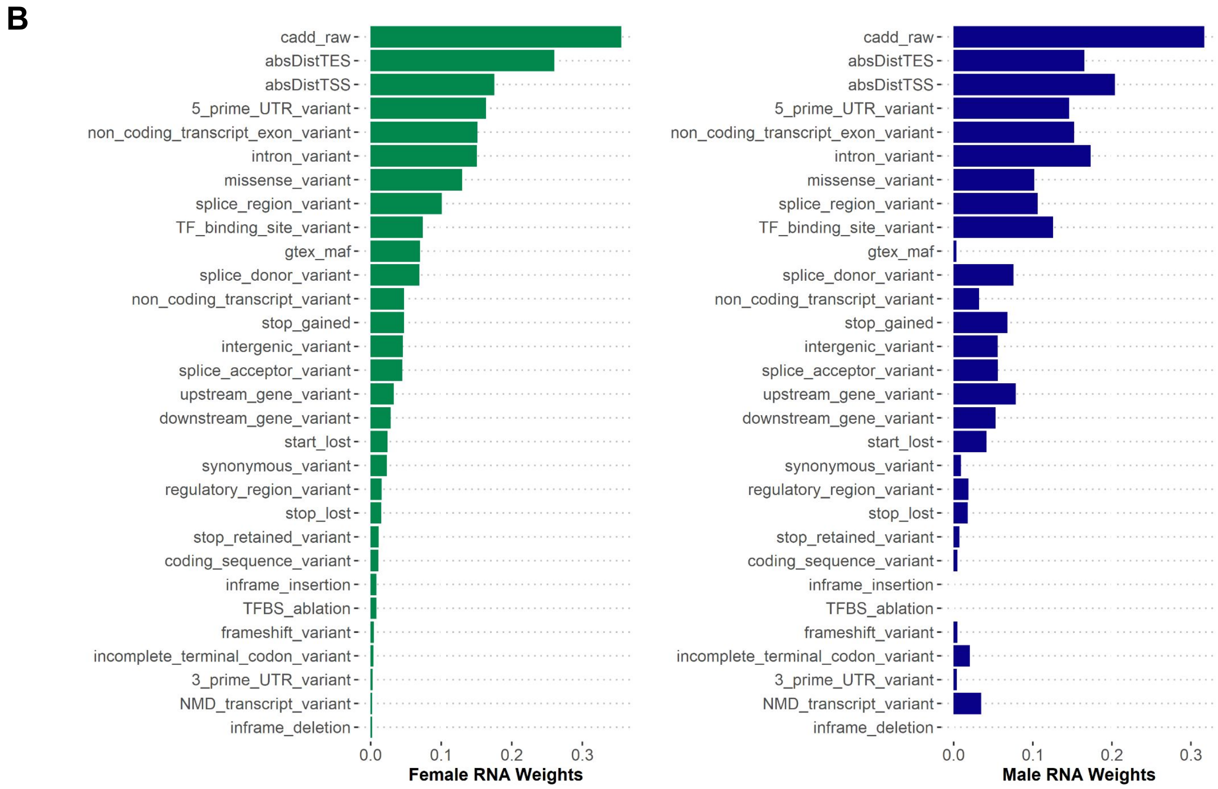

Supplemental Figure 10: RIVER and GAM modeling. a, Precision-recall curve of female (red) and male (blue) specific RIVER and GAM models. **b,** Feature weights of genomic annotations learned from sex-specific RIVER models for females (green) and males (blue), ranked by female-specific weights.

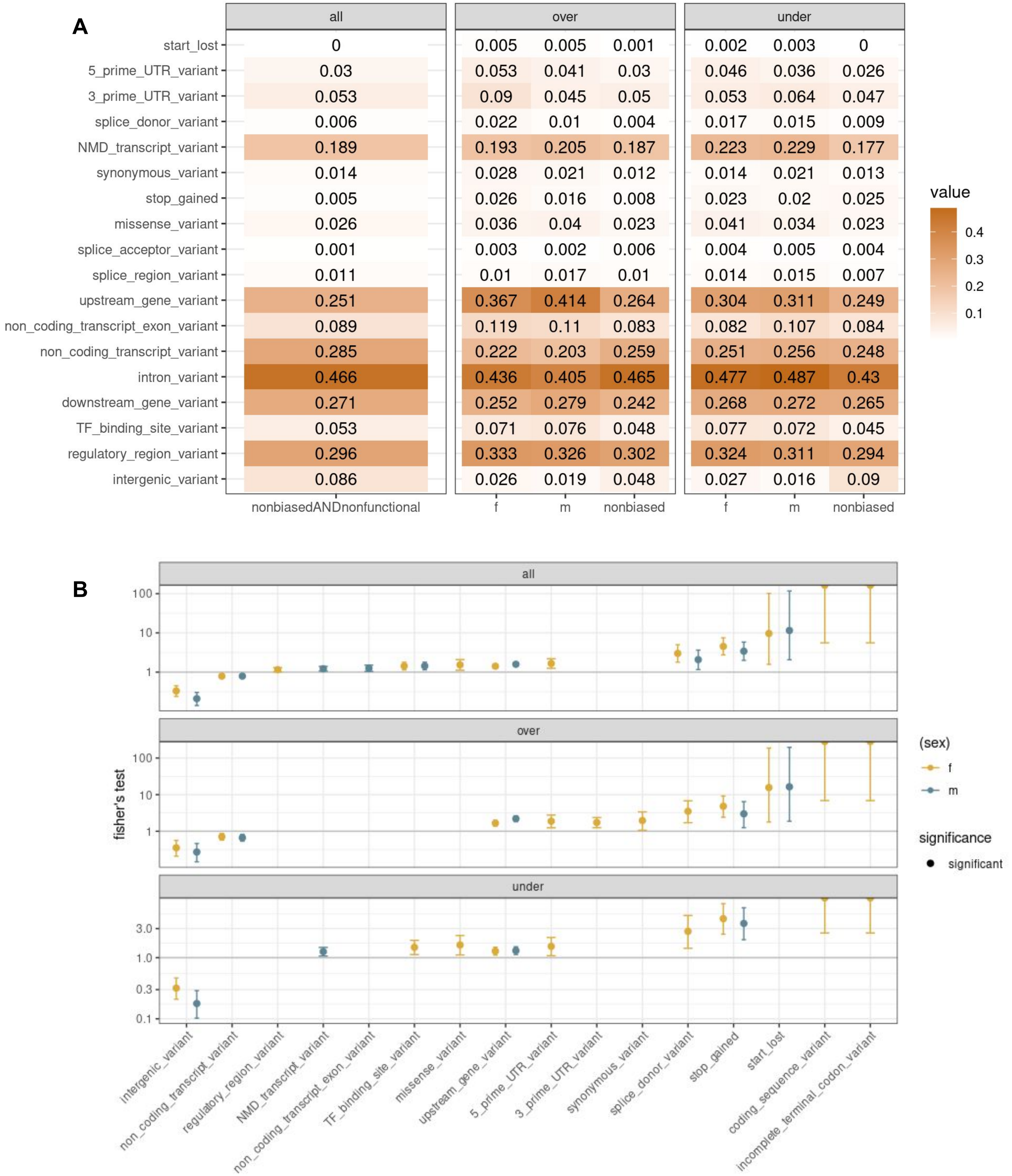

Supplemental Figure 11: a, For each given variant type, the proportion of rare variants that had this annotation (note: variants can have multiple annotations). This is split by over and under expression across female-biased, male-biased, and nonbiased functional rare variants. **b,** Fisher's test for a sex-biased rare variant being in a particular variant category as compared to a non-functional rare variant. This is done for female-biased and male-biased variants, as well as split for predicted direction of effect (over and under).

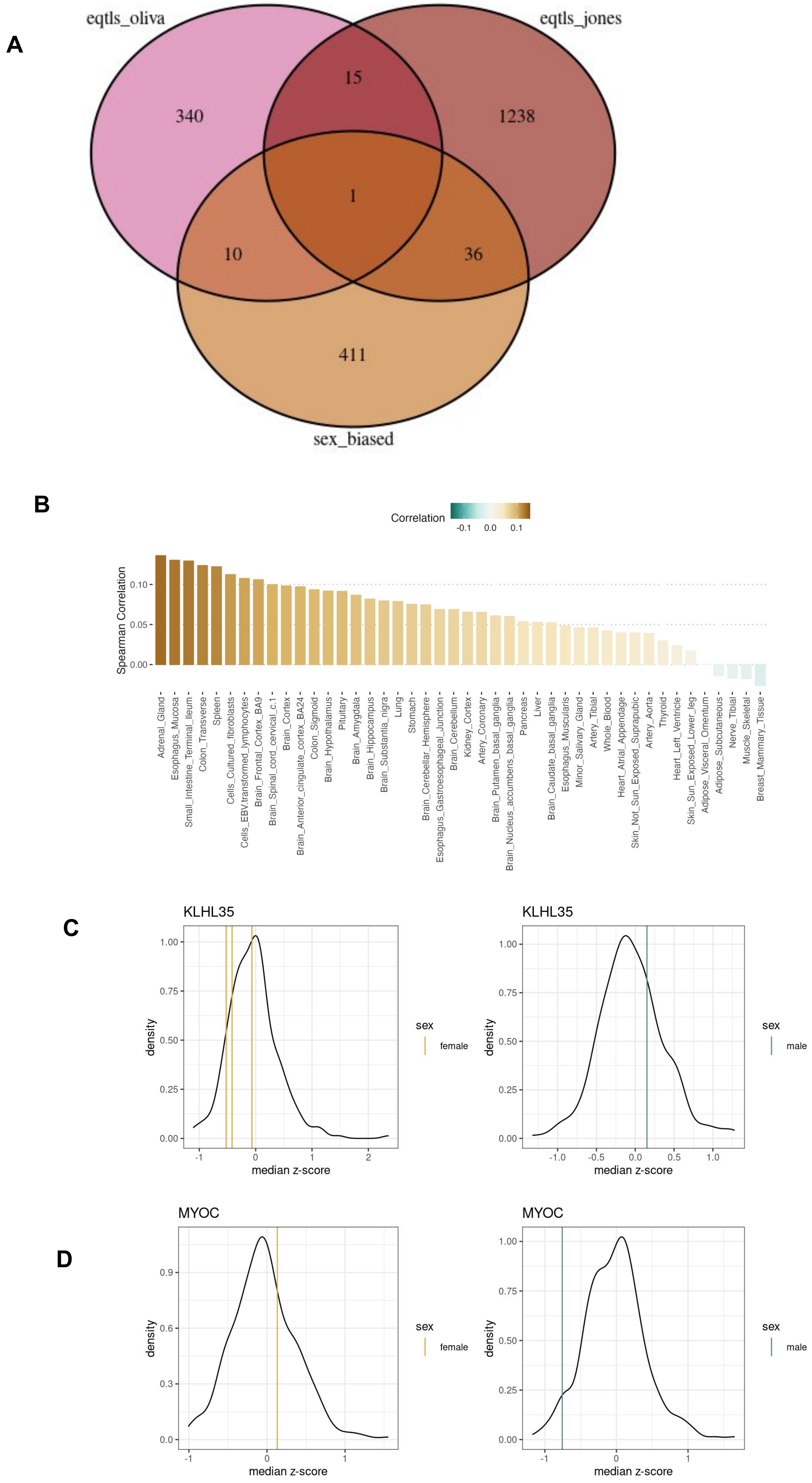
A
eqtls_oliva
eqtls_jones
sex_biased
340
15
1238
1
10
36
411
B
Correlation
-0.1
0.0
0.1
Spearman Correlation
0.10
0.05
0.00
Adrenal_Gland
Esophagus_Mucosa
Small_Intestine_Terminal_Ileum
Colon_Transverse
Spleen
Cells_Cultured_fibroblasts
Cells_EBV.transformed_lymphocytes
Brain_Frontal_Cortex_BA9
Brain_Spinal_cord_cervical_c.1
Brain_Cortex
Brain_Anterior_cingulate_cortex_BA24
Colon_Sigmoid
Brain_Hypothalamus
Pituitary
Brain_Amygdala
Brain_Hippocampus
Brain_Substantia_nigra
Lung
Stomach
Brain_Cerebellar_Hemisphere
Esophagus_Gastroesophageal_Junction
Brain_Cerebellum
Kidney_Cortex
Artery_Coronary
Brain_Putamen_basal_ganglia
Brain_Nucleus_accumbens_basal_ganglia
Pancreas
Liver
Brain_Caudate_basal_ganglia
Esophagus_Muscularis
Minor_Salivary_Gland
Artery_Tibial
Whole_Blood
Heart_Atrial_Appendage
Skin_Not_Sun_Exposed_Suprapubic
Artery_Aorta
Thyroid
Heart_Left_Ventricle
Skin_Sun_Exposed_Lower_leg
Adipose_Visceral_Omentum
Adipose_Subcutaneous
Nerve_Tibial
Muscle_Skeletal
Breast_Mammary_Tissue
C
KLHL35
density
median z-score
sex
female
KLHL35
density
median z-score
sex
male
D
MYOC
density
median z-score
sex
female
MYOC
density
median z-score
sex
male

Supplemental Figure 12: **a**, Overlap of genes that have at least one sex-biased rare variant (orange sex_biased), and genes from Oliva et al 2020 with at least one sex-biased eQTL (pink eqtls_oliva) and genes with at least one sex-biased eQTL from Jones et al (red eqtls_jones). **b,** Spearman correlation of tissue-specific sex-biased gene scores from Oliva et al 2020 with sex-biased rare variants scores. **c,** Distribution of median z-score of KLHL35, with the individuals highlighted who have the predicted sex-antagonistic variant chr11:75430623, split by a female-only distribution and a male-only distribution. **d,** Distribution of median z-score of MYOC, with the individuals highlighted who have the predicted sex-antagonistic variant chr1:171636338, split by a female-only distribution and a male-only distribution.

A

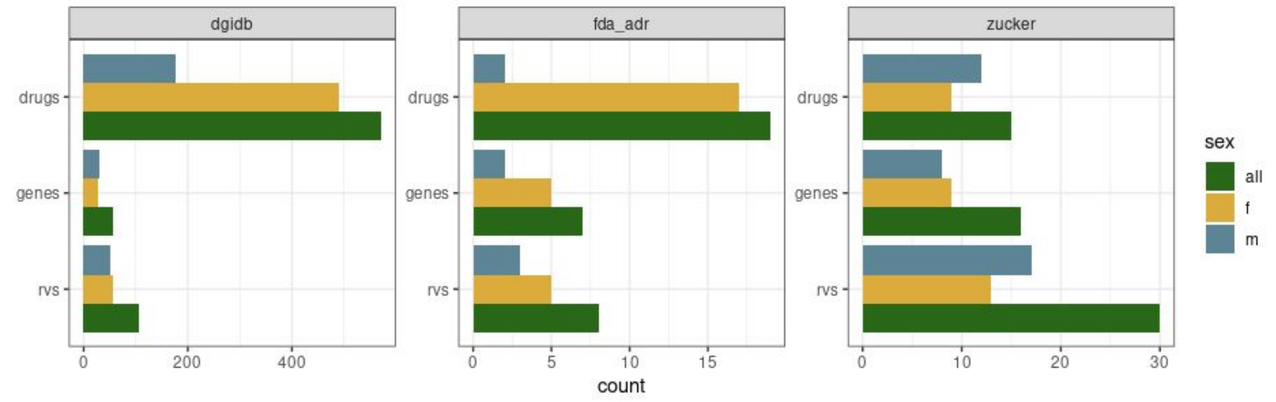

B

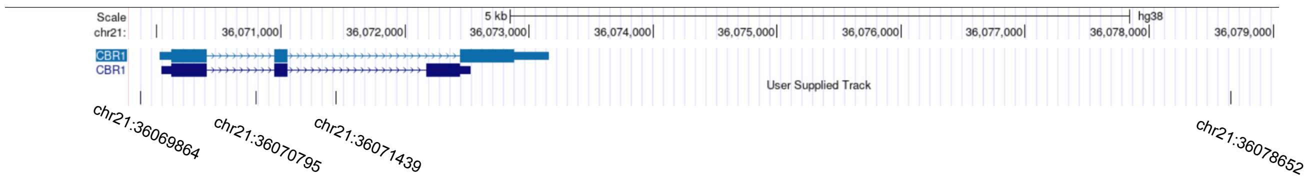

Supplemental Figure 13: a, The number of unique rare variants, genes, and drugs that the functional sex-biased rare variants overlapped with in the DGIdb, FDA adverse drug reaction database and from Zucker et. al. with the different colors representing sex **b,** Edited screenshot of the UCSC genome browser demonstrating the location of sex-biased functional rare variants for the gene CBR1.

A

Rank	Motif	Name	P-value	log P-pvalue	q-value (Benjamini)	# Target Sequences with Motif	% of Targets Sequences with Motif	# Background Sequences with Motif	% of Background Sequences with Motif	Motif File	SVG
1		Six1(Homeobox)/Myoblast-Six1-ChIP-Chip(GSE20150)/Homer	1e-4	-9.450e+00	0.0792	5.0	11.90%	510.1	1.05%	motif file (matrix)	svg
2		EKLF(Zf)/Erythrocyte-Klf1-ChIP-Seq(GSE20478)/Homer	1e-2	-6.442e+00	0.8010	5.0	11.90%	989.7	2.04%	motif file (matrix)	svg
3		Klf4(Zf)/mES-Klf4-ChIP-Seq(GSE11431)/Homer	1e-2	-6.357e+00	0.8010	8.0	19.05%	2647.2	5.45%	motif file (matrix)	svg
4		ATHB53(HB)/col-ATHB53-DAP-Seq(GSE60143)/Homer	1e-2	-5.085e+00	1.0000	4.0	9.52%	850.4	1.75%	motif file (matrix)	svg
5		EHF(ETS)/LoVo-EHF-ChIP-Seq(GSE49402)/Homer	1e-2	-5.022e+00	1.0000	11.0	26.19%	5578.7	11.49%	motif file (matrix)	svg
6		SPL15(SBP)/colamp-SPL15-DAP-Seq(GSE60143)/Homer	1e-2	-4.633e+00	1.0000	8.0	19.05%	3521.2	7.25%	motif file (matrix)	svg

B

Rank	Motif	P-value	log P-pvalue	% of Targets	% of Background	STD(Bg STD)	Best Match/Details	Motif File
1		1e-14	-3.376e+01	4.33%	0.37%	47.2bp (55.8bp)	Hand2(bHLH)/Mesoderm-Hand2-ChIP-Seq(GSE61475)/Homer(0.683) More Information | Similar Motifs Found	motif file (matrix)
2		1e-14	-3.375e+01	1.73%	0.01%	70.7bp (61.2bp)	RBPJ/MA1116.1/Jaspar(0.703) More Information | Similar Motifs Found	motif file (matrix)
3		1e-14	-3.354e+01	5.19%	0.60%	55.0bp (53.4bp)	ZmHOX2a(1)(HD-HOX)/Zea mays/AthaMap(0.649) More Information | Similar Motifs Found	motif file (matrix)
4		1e-13	-3.160e+01	1.52%	0.01%	17.6bp (52.9bp)	RPN4(MacIsaac)/Yeast(0.652) More Information | Similar Motifs Found	motif file (matrix)
5		1e-12	-2.956e+01	2.16%	0.05%	52.5bp (58.1bp)	WIP5(C2H2)/colamp-WIP5-DAP-Seq(GSE60143)/Homer(0.655) More Information | Similar Motifs Found	motif file (matrix)
6		1e-12	-2.821e+01	4.55%	0.56%	46.4bp (52.4bp)	br(var.3)/MA0012.1/Jaspar(0.765) More Information | Similar Motifs Found	motif file (matrix)
7		1e-12	-2.818e+01	1.08%	0.00%	23.8bp (19.4bp)	SNRNP70K(RRM)/Drosophila_melanogaster-RNCMPT00143-PBM/HughesRNA(0.764) More Information | Similar Motifs Found	motif file (matrix)
8		1e-12	-2.818e+01	1.08%	0.00%	44.9bp (0.0bp)	PH0166.1_Six6_2/Jaspar(0.679) More Information | Similar Motifs Found	motif file (matrix)
9		1e-12	-2.816e+01	2.16%	0.06%	40.7bp (49.0bp)	Pit1+1bp(Homeobox)/GCrat-Pit1-ChIP-Seq(GSE58009)/Homer(0.707) More Information | Similar Motifs Found	motif file (matrix)

Supplemental Figure 14: a, Known motif enrichment using HOMER. **b,** *de novo* motif enrichments using HOMER.

A

B

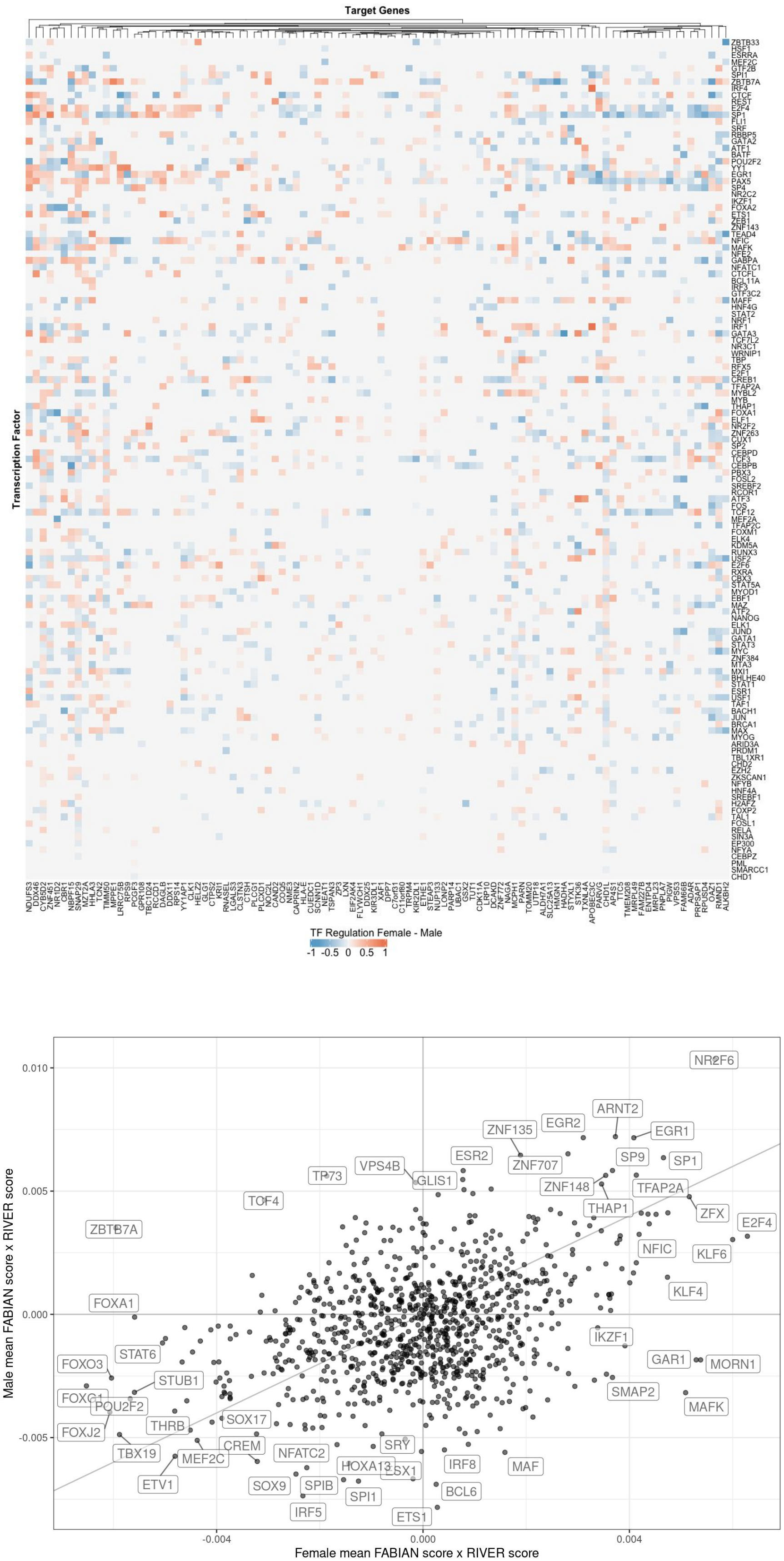

Supplemental Figure 15: a, For each TF-rare variant pair, FABIAN predicts a binding score. The maximum score for a given TF-rare variant pair was collapsed to the gene level. The rows are TFs and columns are genes. The values are the binding score x RIVER score, with the female score subtracted from the male score. **b,** The FABIAN x RIVER collapsed score for same genes in males vs females.
